# An enhanced diabetic retinopathy detection approach using optimized deep learning technique

**DOI:** 10.1038/s41598-026-41998-y

**Published:** 2026-03-23

**Authors:** Saad Mohamed Darwish, Kareema Gumma Milad, Reem Essam El-Din Ibrahim

**Affiliations:** 1 Department of Information Technology, Institute of Graduate Studies and Research, Alexandria University, 21526 Alexandria, Egypt; 2 Faculty of Computer and Data Science, Alexandria University, Alexandria, 5432042 Egypt

**Keywords:** Diabetic Retinopathy, Optimization, Feature Selection, Ensemble Learning, Dynamic Adaptation, Image processing, Literature mining, Predictive medicine

## Abstract

Diabetic Retinopathy (DR) remains a leading cause of vision loss among diabetic patients, underscoring the importance of early detection through reliable retinal imaging analysis. Retinal fundus images are inherently physics-driven, capturing the interactions of light with retinal tissue, including absorption, reflection, and scattering phenomena, which define the intensity and structural patterns critical for diagnosis. However, existing machine learning and optimization approaches for DR screening face challenges in handling the high-dimensional, heterogeneous, and complex physical characteristics of these images. Conventional methods often suffer from suboptimal feature selection, limited generalization, and reduced classification accuracy due to their inability to adaptively exploit image-specific patterns. To address these challenges, this study introduces a Dynamic Grasshopper Optimization Algorithm (DGOA) for feature selection, leveraging its dynamic adaptation capabilities to explore and exploit the physically meaningful feature space effectively. By incorporating adaptive parameter control, DGOA mitigates premature convergence and ensures the selection of the most discriminative features, enhancing model robustness. To further improve classification reliability, an ensemble learning classifier is integrated, combining multiple base models to leverage complementary strengths, reduce overfitting, and maximize predictive performance. The proposed physics-aware AI framework was validated on the EyePACS Retinal Fundus Images dataset, a large and diverse collection of high-resolution images reflecting variations in illumination, contrast, and tissue properties. Comparative experiments with EfficientNetV2S, MGA-CSG, and BWO-DL highlight the advantages of our approach in balancing computational efficiency, generalization, and physically informed feature extraction. The DGOA-Ensemble model achieved an accuracy of 94.6%, F1-score of 0.94, and AUC-ROC of 0.96, demonstrating its effectiveness as a robust, interpretable, and generalizable framework that bridges the gap between physics-based retinal imaging and AI-driven automated DR detection.

## Introduction

 Diabetic retinopathy (DR) is a major complication of diabetes and a leading cause of vision loss among working-age adults worldwide. Early detection is essential, as timely intervention can prevent or delay blindness. In routine clinical practice, DR is evaluated using a combination of ophthalmic imaging modalities, with color fundus photography (CFP) serving as the primary tool for large-scale screening. Additional methods, such as fluorescein angiography (FA) to visualize vascular leakage, optical coherence tomography (OCT) to assess retinal layer integrity and macular edema, and scanning laser ophthalmoscopy (SLO) for high-contrast retinal images, are often employed for comprehensive assessment. Ophthalmologists diagnose DR by identifying characteristic biomarkers including microaneurysms, hemorrhages, hard exudates, cotton-wool spots, intraretinal microvascular abnormalities (IRMA), venous beading, and neovascularization. However, many early lesions are subtle and can be easily overlooked, contributing to variability in manual grading and delayed diagnosis^1,2^.

Retinal fundus imaging is fundamentally a physics-based process, capturing the reflection, absorption, and scattering of light through retinal tissue, including the optic disc, macula, and vascular structures. Variations in illumination, camera optics, and tissue properties introduce complex intensity and contrast patterns that are critical for diagnosing DR, yet traditional computational methods often fail to fully exploit these physically meaningful patterns due to the expansive feature space and heterogeneity of images^3–5^.

Globally, DR affects more than 100 million individuals, and the burden is expected to grow with increasing diabetes prevalence and limited access to retinal specialists in many regions. These challenges highlight the need for scalable and reliable screening solutions. Artificial intelligence (AI) has emerged as a promising tool to support DR detection by enabling rapid image analysis, reducing clinical workload, and providing consistent, objective grading. Importantly, AI algorithms can identify subtle patterns and textural abnormalities that may not be readily apparent to the human eye, offering potential improvements in early detection and triage accuracy. Incorporating AI into DR screening workflows therefore holds significant potential to enhance accessibility, standardization, and diagnostic performance, particularly in high-volume or resource-limited settings.

Existing AI-based DR detection approaches typically fall into three categories: classical machine learning methods using hand-crafted features, convolutional neural networks (CNNs), and hybrid models combining feature selection with classification. Classical models require extensive preprocessing and fail with high-dimensional image data^6,7^, while CNNs provide strong representation learning but demand large labeled datasets and are prone to overfitting^8,9^. Hybrid models aim to improve robustness but often rely on optimization techniques that suffer from premature convergence or weak adaptability to heterogeneous imaging conditions^10^. To provide a clearer overview of the existing approaches, Table 1summarizes the major DR detection methods, their underlying methodologies, strengths, limitations, and representative examples.

Feature selection plays a vital role in improving classification performance and computational efficiency by identifying the most informative features^11,12^. Nevertheless, many existing optimization-based feature selection techniques suffer from parameter sensitivity, premature convergence, and limited adaptability to diverse imaging conditions^13–15^. Therefore, the development of adaptive and efficient optimization algorithms is essential for robust feature selection in retinal image analysis.

A variety of metaheuristic algorithms—such as Genetic Algorithms (GA), Particle Swarm Optimization (PSO), Ant Colony Optimization (ACO), Grey Wolf Optimizer (GWO), and Whale Optimization Algorithm (WOA)—have been explored for medical image analysis, including DR detection^11,12^. Although these methods enhance classification accuracy and reduce feature dimensionality, most require manual parameter tuning and struggle to balance exploration and exploitation effectively^16,17^.

To overcome these limitations, this study employs the Dynamic Grasshopper Optimization Algorithm (DGOA) for optimal feature selection in DR detection. DGOA dynamically adjusts control parameters during iterations, achieving a better balance between exploration and exploitation in high-dimensional feature spaces. Moreover, to further enhance classification accuracy and robustness, ensemble learning techniques—such as bagging, boosting, and stacking—are integrated. These methods combine multiple base learners to reduce overfitting and improve model generalization, leading to more reliable DR detection across diverse datasets^16–18^.

**Table 1:** Summary of common methods for DR detection.

Category	Methodology	Pros	Cons	Representative examples
Traditional Machine Learning (Hand-crafted Features)	Manual extraction of texture, color, morphological, or vessel-based features; classification using SVM, k-NN, Random Forest.	- Simple and interpretable. -Requires smaller datasets. - Low computational cost	-Cannot capture complex patterns -Depends heavily on feature engineering -Limited generalization on diverse datasets	SVM + morphological features; Random Forest + texture features; k-NN + histogram descriptors
Convolutional Neural Networks (CNNs)	End-to-end deep learning models that automatically learn hierarchical features; includes transfer learning and attention models.	-High accuracy -No manual feature extraction -Strong representational power	-Requires large labeled datasets -High computational demands -Less interpretable Sensitive to image quality	InceptionV3 for DR grading; ResNet-based lesion detection; EfficientNet DR classifiers
Hybrid (Feature Selection + Classifier)	Features (hand-crafted or deep) reduced using GA, PSO, ACO, GWO, WOA before classification.	-Removes noisy/irrelevant features. - Improves classifier performance - Works with smaller datasets	- Risk of local optima - Requires tuning optimizer parameters - Moderate computation cost	GA + SVM; PSO + Random Forest; ACO + k-NN; GWO + SVM
Advanced Meta-heuristic Optimization	Uses advanced/chaotic optimizers such as WOA, MVO, SCA, DGOA for high-dimensional feature selection.	- Strong exploration/exploitation - More robust in high-dimensional search - Less premature convergence	- Higher computational complexity - Sensitive to parameters - Variable performance across datasets	WOA + deep features; MVO for DR staging; SCA + RF; DGOA + Ensemble
Ensemble Learning	Combines multiple classifiers using bagging, boosting, or stacking (RF, AdaBoost, XGBoost, meta-learners).	- High robustness and accuracy - Reduces variance and bias - Better generalization	-Less interpretable - More computationally intensive - Requires careful design of base learners	Random Forest; XGBoost + deep features; Stacking (SVM + RF + CNN features)

### Problem statement

DR is a leading cause of vision impairment and blindness among diabetic patients worldwide, necessitating early detection for timely treatment and prevention of disease progression. Despite significant advances in deep learning and image processing, accurate DR detection remains challenging due to the high variability in retinal fundus images caused by differences in resolution, lighting conditions, noise, and patient demographics. Traditional deep learning models and static optimization algorithms often fail to maintain generalizability across diverse datasets, leading to inconsistent diagnostic outcomes. Furthermore, feature extraction and selection from complex, high-dimensional image data continue to pose difficulties, as conventional optimization methods are prone to premature convergence and may fail to identify the most discriminative features. These limitations highlight the need for an adaptive and intelligent detection framework capable of dynamically tuning its internal parameters, improving feature selection, and enhancing classification robustness.

### Research gap

Despite substantial advancements in automated DR detection using deep learning and optimization-based frameworks, several unresolved challenges persist. Current models largely depend on static parameter configurations and fixed optimization settings, which restrict their adaptability to the inherent variability of retinal fundus images—arising from differences in illumination, resolution, and patient demographics. Deep learning architectures and ensemble methods, although achieving high accuracy, often face issues of computational complexity, data imbalance, and limited scalability, making them unsuitable for real-time or resource-constrained clinical environments. Moreover, existing optimization algorithms such as GOA, BDA, and BWO are prone to premature convergence and are highly sensitive to initial parameter settings, leading to suboptimal feature selection and reduced model robustness. Model interpretability and cross-dataset generalization also remain pressing concerns; most frameworks emphasize accuracy at the expense of explainability, limiting clinical trust and transferability across heterogeneous datasets. These limitations underscore the need for an adaptive and dynamic optimization-driven framework that can intelligently tune parameters during training, maintain population diversity, and enhance both feature discrimination and classification stability.

### Study aim

In light of the unresolved challenges identified in existing DR detection models—including static optimization parameters, high computational demands, limited generalization, and insufficient adaptability—there is a clear need for an intelligent detection framework capable of dynamically responding to data variability. This study aims to address that need by developing a DGOA integrated with ensemble learning, forming a unified, adaptive, and high-performance model for automated DR detection. Unlike conventional approaches, the proposed DGOA introduces real-time parameter adjustment mechanisms that adaptively balance exploration and exploitation within the search space, preventing premature convergence and enhancing the discovery of highly discriminative feature subsets. The integration of ensemble learning further strengthens classification robustness by utilizing the complementary strengths of multiple base classifiers, thereby improving accuracy, interpretability, and cross-dataset generalization. This hybrid framework not only enhances detection precision and computational efficiency but also represents a significant step forward toward adaptive, scalable, and clinically reliable DR screening systems.

The remainder of the paper is organized as follows: Sect. 2 reviews recent advancements in automated diabetic retinopathy detection using retinal fundus images. Section 3 details the proposed methodology, including the integration of the dynamic optimization and ensemble learning components. Section 4 presents the experimental evaluation, along with a discussion of results. Finally, Sect. 5 concludes the study and outlines directions for future research.

## Literature review

Automated detection of DR has been extensively studied over the past decade, with both traditional machine learning and deep learning methods demonstrating significant advances^7,19–23^. Early approaches primarily relied on handcrafted feature extraction and rule-based classification techniques using texture, color, and shape descriptors to identify retinal abnormalities^24^. However, such methods often struggled to generalize across varying imaging conditions.

The emergence of deep learning, particularly CNNs, revolutionized DR analysis by enabling automatic feature extraction and improved diagnostic performance^25^. Transfer learning and data augmentation have further enhanced accuracy and generalization, with models such as EfficientNet, Xception, DenseNet, and ResNet widely adopted for DR classification^26–32^. Ensemble and hybrid frameworks integrating multiple CNN architectures or combining deep and traditional machine learning models have also shown superior performance, achieving classification accuracies exceeding 95% on public datasets such as APTOS, EyePACS, and IDRiD^28,30,33–35^.

Recent studies have explored optimization-based and bio-inspired algorithms to improve feature selection and model robustness. Techniques such as the Modified Generative Adversarial-based Crossover Salp Grasshopper (MGA-CSG) approach, Binary Dragonfly Algorithm (BDA), Sine Cosine Algorithm (SCA), and Beluga Whale Optimizer (BWO) have demonstrated strong potential in enhancing convergence speed and feature discrimination in DR classification tasks^29,34,36^. Similarly, hybrid models integrating CNNs with heuristic optimizers like Bacterial Foraging Optimization (BFO) and GWO achieved high performance while reducing feature dimensionality^37,38^.

Moreover, recent works have focused on model interpretability and real-world applicability. Explainable AI techniques such as Grad-CAM and SHAP have been employed to visualize lesion localization, increasing the clinical trustworthiness of DR detection systems^32,39^. The integration of DR detection frameworks with Internet of Things (IoT) platforms has also been explored for remote and resource-limited clinical environments^36^.

Khalili Pour et al.^40^ present an AutoML-based approach for distinguishing proliferative from non-proliferative diabetic retinopathy using OCTA vascular density maps, offering strong clinical interpretability through vascular biomarkers but limited scalability and reliance on expensive imaging modalities. In contrast, Rezaee and Farnami^41^ propose a CNN–Transformer fusion model for DR severity classification from retinal images, achieving higher accuracy and better multi-stage classification through deep feature learning, albeit with greater computational cost and reduced interpretability. Together, these studies reflect the trade-off between interpretability and performance in modern DR classification methods.

Recent studies have further advanced diabetic retinopathy detection by integrating deep learning, feature decomposition, and hybrid optimization strategies. Bilal et al.^42^ introduced a transfer learning framework combined with a U-Net architecture for automatic DR lesion segmentation and detection, demonstrating improved localization accuracy in fundus images. In a subsequent work, Bilal et al.^43^ proposed a CNN-SVD-enhanced Support Vector Machine, where deep features were decomposed using Singular Value Decomposition to reduce redundancy before classification, achieving robust performance for vision-threatening DR detection. Similarly, DeepSVDNet^44^ utilized deep feature extraction followed by dimensionality reduction to enhance classification stability while mitigating overfitting. Comprehensive reviews by Ikram et al.^45^ highlighted current trends in fundus-based DR grading, emphasizing the growing adoption of hybrid CNN architectures, attention mechanisms, and explainability tools, while also underscoring persistent challenges related to domain shift and clinical validation. More recently, ResViT FusionNet^46^ combined CNN and Vision Transformer representations to improve feature fusion and interpretability, offering enhanced performance at the expense of increased computational complexity. Additionally, Khan et al.^47^ presented a hybrid genetic grey wolf optimization scheme integrated with kernel extreme learning machines, demonstrating the effectiveness of evolutionary optimization for feature selection and classification accuracy. Collectively, these studies illustrate a clear shift toward hybrid, optimization-assisted, and explainability-driven frameworks, while also revealing ongoing challenges in scalability, generalization, and deployment readiness.

### Discussion

Nevertheless, the implementation and generalization of these methods continue to face notable challenges across several dimensions^30,33,35,48^. A primary difficulty lies in the dependence on large, high-quality annotated datasets. Deep learning models such as ResNet, DenseNet, and EfficientNet have achieved high accuracy^27–32^, yet their performance is often constrained by data scarcity, class imbalance, and variability in image quality. Retinal fundus images captured under different conditions exhibit inconsistent illumination, contrast, and noise levels, which may hinder robust feature extraction. Although data augmentation and transfer learning techniques^28,30,32,35^ have been introduced to address these issues, they only partially mitigate the problem, and synthetic data generation methods risk introducing artificial biases.

Another persistent challenge involves the computational complexity and scalability of existing approaches. Ensemble models and hybrid deep learning frameworks^28,30,32,35^ often require substantial processing power and training time, limiting their suitability for real-time clinical deployment, particularly in low-resource settings. Optimization-based techniques, including the GOA, BDA, and Beluga Whale Optimizer (BWO)^29,34,36^, have been employed to enhance feature selection and convergence speed. However, these algorithms are often sensitive to initial parameter settings and may experience premature convergence, which restricts their adaptability in complex, high-dimensional feature spaces.

A further area of concern relates to model interpretability and clinical reliability. Although explainable AI approaches such as Grad-CAM and SHAP^32,39^ have been proposed to visualize model decisions, their integration into DR detection frameworks remains limited. The majority of existing models prioritize accuracy metrics over interpretability, posing challenges for clinical validation and practitioner trust. For medical image analysis, interpretability is essential not only for transparency but also for identifying cases of model uncertainty and misclassification.

Additionally, generalizability across diverse datasets remains an unresolved issue. Models trained on specific datasets, such as APTOS, EyePACS, or IDRiD, may underperform when applied to images from different populations, devices, or imaging conditions^30,33,35,48^. This lack of cross-dataset robustness limits their real-world applicability. While ensemble and hybrid approaches improve generalization to some extent, they also introduce additional layers of model complexity and parameter tuning, increasing implementation difficulty.

Finally, the balance between diagnostic accuracy and computational efficiency continues to pose a significant trade-off. Highly accurate deep architectures and optimization-based models tend to demand extensive hardware resources and longer training cycles, which can impede their integration into portable or cloud-based diagnostic systems. Achieving an equilibrium between these competing requirements remains one of the most pressing challenges in automated DR detection research.

Recent studies have increasingly adopted hybrid frameworks that combine deep learning, feature decomposition, optimization, and explainability to improve diabetic retinopathy detection. Transfer learning and U-Net–based models enhance lesion localization but remain dependent on high-quality annotations and controlled imaging conditions^42^. Feature decomposition approaches such as CNN-SVD and DeepSVDNet reduce redundancy and improve classification stability, yet their fixed decomposition strategies may limit adaptability across heterogeneous datasets^43,44^. Fusion architectures integrating CNNs and Vision Transformers achieve richer feature representations and improved interpretability, though at the cost of increased computational complexity and reduced deployment feasibility^46^. Optimization-driven classifiers, including genetic grey wolf optimization combined with kernel extreme learning machines, demonstrate the effectiveness of evolutionary feature selection but introduce sensitivity to parameter initialization and convergence behavior^47^. Systematic reviews further highlight persistent challenges related to domain shift, cross-device variability, and insufficient external clinical validation despite notable performance gains^45^. Collectively, these works underscore significant progress in DR detection while revealing ongoing limitations in scalability, generalization, and real-world applicability.

Despite the progress achieved by previous studies in automated DR detection, several limitations in the existing literature remain. Many prior approaches rely heavily on large, high-quality annotated datasets, making them vulnerable to data scarcity, class imbalance, and variability in image quality. Additionally, models trained on specific datasets often fail with domain shift, performing suboptimally when applied to images from different populations, devices, or imaging conditions. Similarly, cross-device variability in fundus cameras, image resolution, and acquisition conditions could impact feature distributions and reduce generalizability in new clinical environments. Furthermore, although extensive cross-validation and multiple train-test splits were employed to assess performance, additional external clinical validation on independent datasets is necessary to confirm the framework’s reliability and applicability in real-world settings. Addressing these limitations in future work—through multi-center data collection, domain adaptation techniques, and prospective clinical studies—will be critical to ensure the safe and effective deployment of automated DR detection systems.

The novelty of this study lies not in introducing an entirely new optimization paradigm, but in systematically advancing adaptive feature selection for diabetic retinopathy detection through a dynamically controlled Grasshopper Optimization framework integrated with ensemble learning. While previous studies have explored improved or hybrid metaheuristic algorithms—including modified GOA, BDA, BWO, SCA, and MGA-CSG—for retinal image analysis, their adaptability is typically limited to predefined parameter schedules, heuristic decay functions, or task-specific tuning strategies. Such approaches, although effective in accelerating convergence, do not consistently adapt to evolving feature distributions or explicitly address the exploration–exploitation imbalance in high-dimensional retinal feature spaces.

In contrast, this work introduces a DGOA that performs continuous, iteration-wise parameter adaptation during feature selection. Rather than relying on static or semi-static control coefficients, the proposed DGOA dynamically regulates grasshopper interaction forces to maintain population diversity, mitigate premature convergence, and enhance the identification of discriminative retinal features under heterogeneous imaging conditions. This dynamic adaptation is explicitly designed for feature-level optimization, distinguishing it from prior studies that primarily focus on network-level tuning or convergence acceleration.

Furthermore, the proposed framework uniquely couples DGOA-driven feature selection with ensemble learning, utilizing the complementary strengths of multiple classifiers to improve robustness, generalization, and classification stability across diverse datasets. This integration addresses a critical limitation in existing adaptive metaheuristic approaches, which often optimize feature subsets without systematically enhancing downstream classification reliability.

## Methodology

Automated DR detection involves not only computational modeling but also the analysis of retinal images that are inherently governed by optical and physical principles. Fundus images capture light interactions with retinal tissue, including absorption, reflection, and scattering phenomena that produce the structural and contrast features critical for clinical diagnosis. The proposed framework integrates these physics-informed imaging characteristics with advanced AI techniques, specifically DGOA and ensemble learning, to adaptively extract discriminative features for accurate DR classification. By bridging the gap between physics-based retinal imaging and machine learning-driven analysis, this study offers an interdisciplinary approach that aligns with the journal’s scope, demonstrating both the scientific rigor of physical modeling and the practical utility of AI in biomedical applications.

The proposed DR detection framework employs a two-level stacked ensemble learning architecture. In the first level, multiple heterogeneous base classifiers—SVM, BN, and Decision Tree DT—are trained independently using the optimal feature subset selected by the DGOA algorithm. In the second level, a gradient boosting decision tree model (LightGBM) is used as a meta-classifier to fuse the probabilistic outputs of the base classifiers and generate the final prediction. This stacked design enables the system to exploit complementary decision patterns learned by different classifiers while maintaining computational efficiency and interpretability.

- **Base Classifier Layer (Level 1)**: Each base classifier receives the same DGOA-selected feature vectors as input and is trained independently to predict DR class probabilities. The SVM captures discriminative margins in high-dimensional feature space, the Bayesian Network models probabilistic dependencies among features, and the Decision Tree learns rule-based hierarchical feature splits. Instead of producing hard class labels, all base classifiers output calibrated class probabilities. These probability vectors form the intermediate representation used for ensemble fusion.
- **Meta-Classifier Layer (Level 2)**: The probability outputs from the base classifiers are concatenated to form a low-dimensional meta-feature vector, which serves as the input to the LightGBM model. LightGBM operates as a gradient boosting meta-learner, sequentially constructing decision trees that minimize classification loss by correcting errors made in previous iterations. By learning optimal nonlinear combinations of base classifier outputs, LightGBM enhances robustness, improves minority class recognition, and mitigates individual model biases.
- **Final Prediction and Decision Making**: The trained LightGBM meta-classifier outputs final prediction probabilities, which are converted into DR class labels using predefined thresholds for binary or multi-class classification. This structured fusion mechanism ensures that the final decision reflects complementary evidence from all base classifiers while maintaining a clear and interpretable ensemble workflow.

The proposed approach begins with a data preprocessing pipeline to enhance the quality and consistency of retinal fundus images, addressing common challenges such as lighting variations, blur, and imaging artifacts. Images are first resized to a uniform resolution and converted to grayscale using a weighted RGB-to-gray transformation, simplifying the input without losing important structural information. Missing data and noise are corrected using imputation techniques like median filtering, and contrast enhancement is applied via histogram equalization to better expose retinal features such as microaneurysms, exudates, and blood vessels. These preprocessing steps are critical to ensure that subsequent feature extraction processes operate on clean and normalized input, thereby enhancing the reliability and accuracy of the model.

**Fig. 1:**
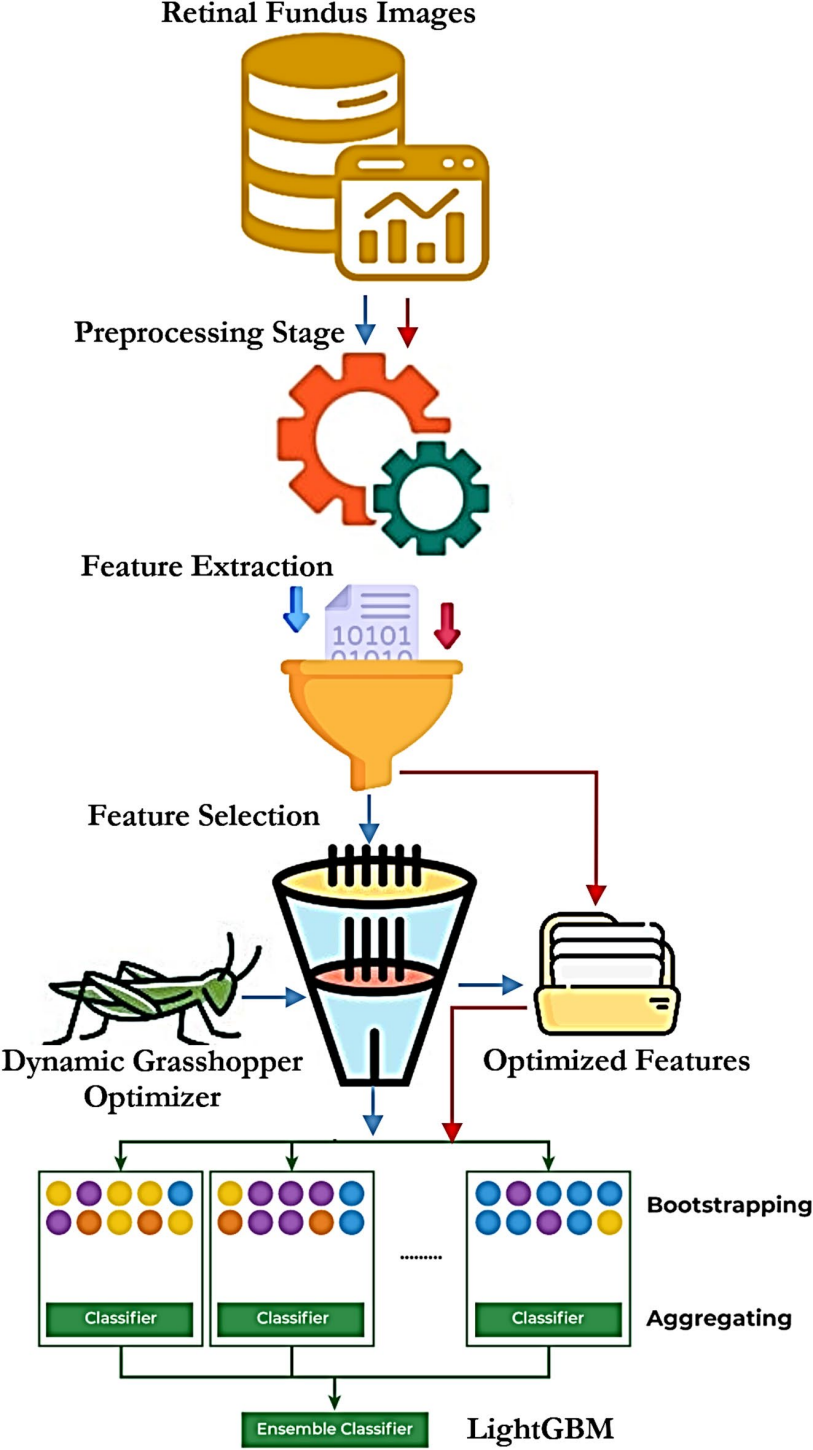
The schematic diagram of the suggested model.

Following preprocessing, a CNN is employed to automatically extract deep hierarchical features that capture complex visual patterns associated with DR. These features are further optimized using DGOA, which efficiently selects the most informative features by exploring large, discrete search spaces and removing redundancy to improve generalization and reduce overfitting. For classification, the system utilizes a stacked ensemble model with SVM, Bayesian Networks, and Decision Trees as base learners. The outputs (class scores) of these learners are fed into LightGBM as a meta-learner. LightGBM, a highly efficient gradient boosting framework, combines the base learners’ outputs using its leaf-wise histogram-based tree growth strategy, which selects the leaf with the maximum delta loss for splitting. This method leads to faster convergence and higher accuracy than traditional level-wise algorithms. This architecture utilizes the strengths of each base learner—SVM’s margin maximization, Bayesian Network’s probabilistic modeling, and Decision Trees’ rule-based logic—while LightGBM ensures optimal final classification through its robust ensemble boosting mechanism. Here is a detailed explanation of how these models function together, with Fig. 1 illustrating the overall process.

### Data acquisition

**Fig. 2:**
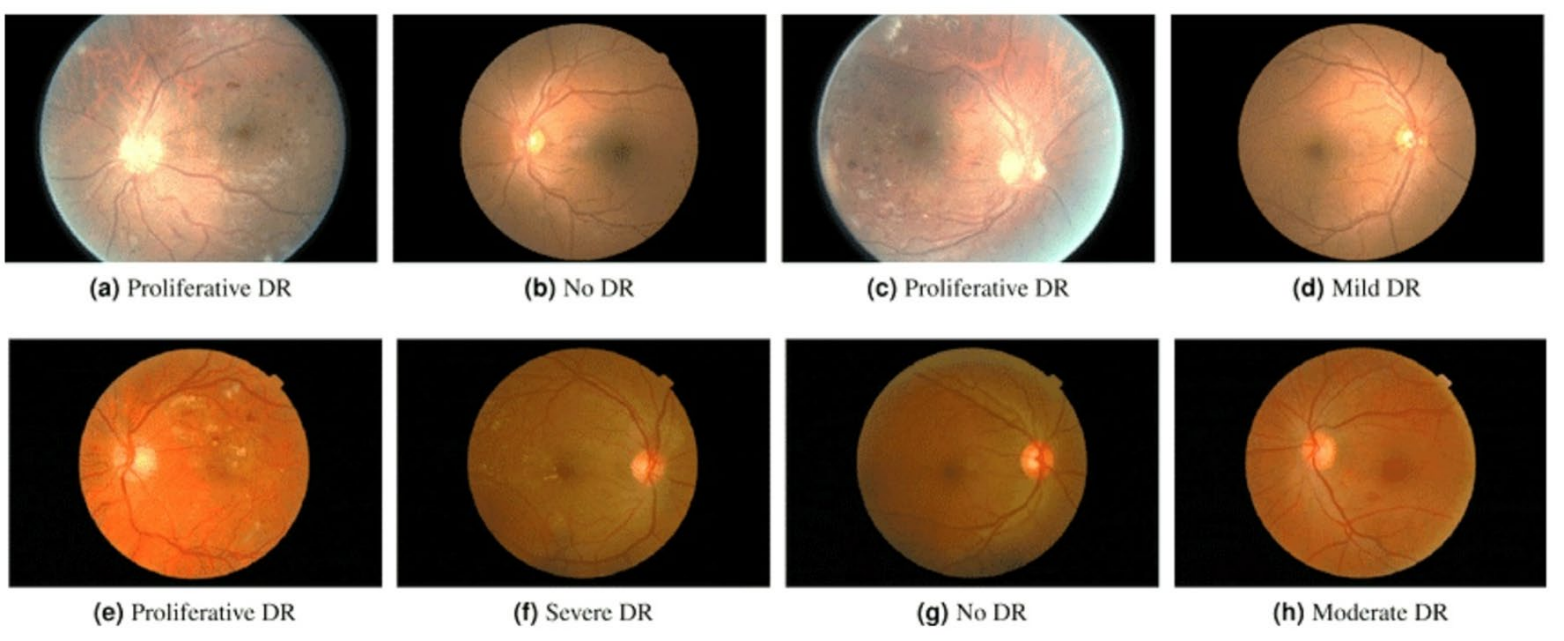
The top row displays sample images from the Kaggle-EyePACS dataset, while the bottom row features images from the Messidor-2 dataset, along with their respective DR grades. The first row highlights the two types of fundus images found in the Kaggle-EyePACS dataset: images where the circular disk of the eye is fully visible (right column) and those where the circular disk is partially cropped (left column).

Let the input dataset consist of $N$retinal fundus images:

1 $$D={\left\{\left\{{I}_{i},{y}_{i}\right\}\right\}}_{i=1}^{N}$$

in which ${I}_{i}\in{\mathbb{R}}^{H\times{W}\times{C}}$is the $i$-th retinal image with height$H$, width $W$, and $C$ color channels. ${y}_{i}\in\left\{\mathrm{0,1},2,\dots..,K-1\right\}$ is the corresponding label, indicating the severity of DR (e.g.,$K=2$ for binary classification or $K>2$ for multi-class classification). The Kaggle-EyePACS dataset retinal fundus images dataset on Kaggle (see Fig. 2) is a comprehensive collection of high-quality images of the human retina, captured using fundus cameras for the detection and classification of DR. The dataset consists of thousands of annotated images in JPEG format, with file sizes ranging from 500 KB to several megabytes, depending on the resolution. The retinal fundus images in the dataset typically range from 1,000 to 2,000 pixels in width and are captured at varying resolutions, covering key retinal structures such as the optic disc, macula, and blood vessels. These images are categorized based on the clinically recognized stages of diabetic retinopathy, comprising two main types: Non-Proliferative Diabetic Retinopathy (NPDR) and Proliferative Diabetic Retinopathy (PDR). NPDR is further divided into mild, moderate, and severe stages, while PDR represents the advanced stage of the disease characterized by abnormal neovascularization. Accordingly, the dataset includes labels such as ‘No DR’, ‘Mild NPDR’, ‘Moderate NPDR’, ‘Severe NPDR’, and ‘Early PDR’ to capture these clinically meaningful distinctions. The dataset consists of over 35,000 retinal fundus images obtained from various fundus camera models, introducing variability in image quality, illumination, and contrast. Some images contain artifacts such as reflections, blurring, or motion-induced distortions. To address these inconsistencies and improve model performance, preprocessing steps including normalization, resizing, and contrast enhancement are applied prior to training the DR detection models^1,4^.

### Preprocessing data

During preprocessing, all retinal fundus images were converted to grayscale to reduce the influence of illumination variations, camera differences, and non-informative color cues that can introduce noise into the learning process. In DR detection, the most diagnostically relevant information—such as microaneurysms, hemorrhages, exudates, and vessel abnormalities—resides primarily in the intensity and texture domains rather than in color information. The grayscale transformation simplifies the feature space by focusing on structural and contrast-based patterns, thereby reducing computational complexity and improving convergence stability. Moreover, grayscale conversion helps minimize overfitting by eliminating redundant color channels that may not contribute significantly to lesion localization or vascular morphology recognition. This preprocessing step ensures that the model concentrates on clinically relevant features, such as vessel geometry and localized intensity variations that are strongly associated with the progression of DR. This preprocessing step ensures that images are standardized and optimized for feature extraction^2,3^.

**Fig. 3:**
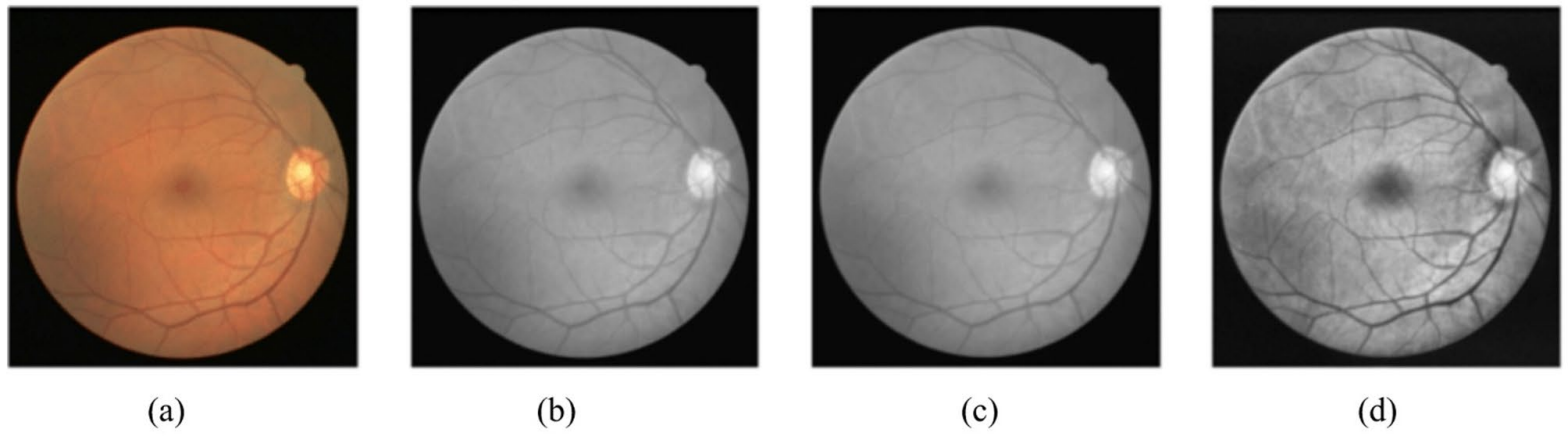
Illustration of the preprocessing. (**a**) Original fundus image, (**b**) Grayscale image, (**c**) Median filtering image, and (**d**) Application of histogram equalization to obtain the final preprocessed image.

- First, raw images ${I}_{raw}$​ are resized to a uniform dimension $\left({H}^{{\prime}},{W}^{{\prime}}\right)$ using the operation ${I}_{k}^{{\prime}}=resize({I}_{k},H{\prime},W{\prime})$ where ${I}_{k}^{{\prime}}\in{\mathbb{R}}^{{H}^{{\prime}}\times{w}^{{\prime}}\times{C}^{{\prime}}}$with ${C}^{{\prime}}=3$ for color or${C}^{{\prime}}=1$ for grayscale.
- Next, resized images ${I}_{resized}$ are converted to grayscale using the formula${I}_{k}^{{\prime}{\prime}}(x,y)=0.2989\cdot{R}(x,y)+0.5870\cdot{G}(x,y)+0.1140\cdot{B}(x,y)$, where $R,G,andB$ represent the red, green, and blue channels.
- Any missing data in ${I}_{gray}$​ is addressed using imputation technique (median filling), resulting in cleaned images${I}_{\mathrm{c}\mathrm{l}\mathrm{e}\mathrm{a}\mathrm{n}\mathrm{e}\mathrm{d}}$​.
- Finally, enhancement methods like histogram equalization or filtering are applied to improve image quality, producing enhanced images${I}_{\mathrm{e}\mathrm{n}\mathrm{h}\mathrm{a}\mathrm{n}\mathrm{c}\mathrm{e}\mathrm{d}}$ (see Fig. 3).

### Feature extraction

The most suitable feature extraction technique for DR detection in retinal fundus images, according to the literature, is Deep Learning-based Feature Extraction using CNNs^49^. CNNs have proven highly effective because they automatically learn hierarchical features directly from the input images without requiring manual intervention^50^. These features range from low-level attributes like edges and textures to high-level patterns such as Microaneurysm Density, Exudate Presence, and Hemorrhage Spread. The ability to adaptively capture both local and global structures within the retinal fundus image ensures that CNNs effectively handle the complex and diverse manifestations of DR^51^. Moreover, advanced CNN architecture like EfficientNet, combined with transfer learning, have demonstrated exceptional performance in identifying subtle lesions and accurately grading the severity of DR, often surpassing traditional feature extraction methods in sensitivity and specificity^52,53^.

The average length of the feature vector extracted by a CNN for DR detection in retinal fundus images varies based on the architecture and the layer used for feature extraction. When features are obtained from the fully connected (FC) layer in pre-trained models like ResNet, VGG, or EfficientNet, the vector length is typically 2,048 for ResNet50, 4,096 for VGG16, and 1,280 for EfficientNet-B0, representing compact, high-level representations of the image. In our case, we utilize EfficientNet-B0 for feature extraction. EfficientNet is the most efficient configuration for tasks like DR detection in retinal fundus images due to its compound scaling method, which optimally balances network depth, width, and resolution to achieve high performance with minimal computational cost^52,53^.

In this study, EfficientNet-B0 was employed as the deep learning-based feature extractor for retinal fundus images. The model was pre-trained on the ImageNet dataset and used without any architectural modifications. Features were extracted from the final FC layer, resulting in a feature vector of length 1,280 for each image. EfficientNet-B0 was selected due to its optimal balance of accuracy and efficiency, achieved through compound scaling of depth, width, and resolution. The implementation was carried out using the TensorFlow framework (version 2.16.1) and Keras, leveraging built-in pre-trained weights to avoid the need for extensive model training. This approach enables the extraction of compact, high-level representations that capture both local and global structures relevant to diabetic retinopathy detection. See^54,55^ for more details.

However, since EfficientNet B0 is pretrained on RGB ImageNet images and expects a three-channel input, direct feeding of single-channel grayscale images would disrupt the model’s pretrained convolutional filter structure. To maintain architectural consistency while using pretrained weights, each grayscale image was replicated across three channels, producing a synthetic RGB representation of size 224 × 224 × 3. This replication ensures compatibility with the pretrained model while allowing the convolutional filters to process luminance-based texture variations across all channels. Although the replicated channels are identical, the convolutional kernels—especially in the early layers—respond to subtle gradient transitions and intensity contrasts that capture fine retinal structures such as microaneurysms and vessel intersections. This approach retains the benefit of transfer learning without retraining the entire network from scratch.

In our case, after replicating each grayscale retinal fundus image across three channels, the resulting synthetic RGB images were further preprocessed to match the input distribution of the ImageNet-pretrained EfficientNet-B0 model. Specifically, all images were resized to 224 × 224 × 3 to ensure a fixed spatial resolution compatible with the network architecture, and pixel intensities were scaled to a standardized range to improve numerical stability during optimization. Subsequently, channel-wise normalization using the standard ImageNet mean and standard deviation was applied to align the input feature distribution with that observed during pretraining, thereby reducing domain shift and facilitating effective transfer learning. These preprocessing steps enhance training stability, accelerate convergence, and improve the overall classification performance of the EfficientNet-B0 model.

Additionally, fine-tuning was performed on the final layers of EfficientNet-B0 to adapt the model’s pretrained color-sensitive filters to the grayscale retinal domain. Specifically, the last two convolutional blocks (MBConv6 blocks) preceding the classification head were unfrozen, while the earlier feature extraction layers remained frozen to preserve general low-level edge and shape detectors learned during ImageNet pretraining. The fine-tuning process involved retraining the classification head and the unfrozen convolutional blocks for 30 epochs using a batch size of 32, the Adam optimizer, and a low learning rate of${10}^{-4}$ to enable gradual weight updates without disrupting pretrained spatial hierarchies. These hyperparameter values were selected to ensure stable convergence while minimizing overfitting, given the limited size and grayscale nature of the retinal dataset^11,13^.

Through this controlled adaptation, deeper color-dependent filters were recalibrated to emphasize intensity gradients, vessel continuity, lesion texture, and macular density variations—features that are critical for distinguishing between different DR severity levels. Overall, this targeted fine-tuning strategy enhances sensitivity to structural and textural cues while suppressing reliance on irrelevant chromatic information, resulting in a robust and computationally efficient feature extractor optimized for grayscale-based diabetic retinopathy detection.

### Feature selection using DGOA

The DGOA is employed in this study to perform adaptive and robust feature selection for retinal fundus image analysis. DGOA is an enhanced version of the classical GOA, designed to address limitations such as premature convergence, slow exploration of the search space, and sensitivity to parameter initialization—issues that are particularly critical in high-dimensional medical imaging tasks. The original GOA models grasshopper behavior through three key components: (1) social interaction forces between individuals, (2) gravitational attraction, and (3) wind advection, which together generate position updates that balance exploration and exploitation. While this mechanism allows GOA to search nonlinear objective functions effectively, its static control parameters often result in reduced search diversity as iterations progress^16^.

To overcome these challenges, DGOA introduces dynamic parameter adaptation, allowing the algorithm to adjust its exploration–exploitation balance based on the current generation and population state. Specifically, the comfort zone coefficient is updated using a nonlinear decay strategy, enabling broader exploration in early iterations and progressively stronger exploitation as convergence nears. This dynamic adjustment prevents population stagnation, improves global search capability, and reduces the risk of becoming trapped in local optima.

During feature selection, each grasshopper represents a candidate subset of features encoded as a binary vector. The fitness function evaluates the quality of each subset by combining classification accuracy and feature reduction rate, ensuring that the selected features are both discriminative and computationally efficient. Through iterative updates using DGOA operators, irrelevant and redundant features are gradually removed while the most informative retinal characteristics are retained. By integrating DGOA into the proposed framework, the feature selection process becomes more stable, adaptive, and effective across heterogeneous DR datasets with varying image quality. This results in a more compact and robust feature set that enhances downstream ensemble classification performance.

The mathematical formulation of a grasshopper’s position update in a D-dimensional search space is given by^17^:

2 $${X}_{i}=\sum_{j=1,j\ne{i}}^{{P}_{S}}s\left({d}_{ij}\right){\widehat{d}}_{ij}+G+W$$

3 $${d}_{ij}=\parallel{X}_{j}-{X}_{i}\parallel,{\widehat{d}}_{ij}=\frac{{X}_{j}-{X}_{i}}{{d}_{ij}}$$

where, ${X}_{i}$ is the position vector of the i-th grasshopper, ${P}_{S}$ is the population size, ${d}_{ij}$ is the Euclidean distance between grasshopper $i$ and $j$. ${\widehat{d}}_{ij}$ is the unit vector pointing from $i$ to $j$. $G$ and $W$ represents the influence of gravity and wind direction, respectively, which guide the agents towards the global best position. $s\left(d\right)$ is a nonlinear social force function defined as^17^:

4 $$s\left(d\right)=f{e}^{\raisebox{1ex}{$d$}\!\left/\!\raisebox{-1ex}{$l$}\right.}-{e}^{-d}$$

$f$ is the intensity of attraction and $l$ is the attractive length scale.

However, one of the primary limitations of the classical GOA is the use of static control parameters, which can restrict the optimizer’s ability to escape local optima and slow convergence. To overcome this, DGOA introduces dynamic control parameters that evolve during the search process. These parameters are typically modulated using a linear or nonlinear decay strategy based on the iteration number. For instance, the coefficient $c$, which controls the intensity of the social forces, is dynamically updated as^17^:

5 $$C={C}_{max}-\left(\frac{{C}_{max}-{C}_{min}}{T}\right)\times{t}$$

​ ${C}_{max}$ and ${C}_{min}$are the initial and final values of the control coefficient.$t$ is the current iteration, $T$ is the maximum number of iterations. In DGOA, the key to achieving a balanced trade-off lies in adapting the social interaction strength (control coefficient) over time. At the beginning of the optimization, the algorithm favors exploration by allowing agents (grasshoppers) to move more freely and explore diverse regions of the search space. This is controlled by a higher value of the control parameter $c$, promoting wide-range movement and avoiding premature convergence. As the number of iterations progresses, the parameter $c$ is gradually decreased (typically using a linear or exponential decay function), thereby reducing the movement range of grasshoppers. This transition encourages exploitation, where the agents start to concentrate around promising areas identified earlier in the search. Moreover, the interaction model of grasshoppers in DGOA considers both attraction and repulsion mechanisms, allowing it to handle complex decision boundaries and nonlinear feature interactions more effectively than static optimization methods^18^.

The problem representation begins with a dataset $X$, consisting of features extracted from retinal fundus images, denoted as $X=\{{x}_{1},{x}_{2},\dots,{x}_{N}\}$ where each sample ${x}_{i}\in{\mathbb{R}}^{m}$ represents one image, $N$ is the total number of images, and $m$ is the total number of features. The goal is to derive a reduced feature subset ${X}_{s}\subseteq{X}$, represented as${X}_{s}=\{{x}_{s1},{x}_{s2},\dots,{x}_{sk}\}$, where $k<m$, ensuring that the selected features maximize the detection accuracy of DR while discarding redundant or irrelevant information.

*Step 1: Initialization*.

Grasshopper Population: $P=\{p{}_{1},{p}_{2},\dots,{p}_{N}\}$: Randomly initialize positions of $N$ grasshoppers in the feature space, where each position corresponds to a feature subset. $p{}_{i}=\{p{}_{i1},p{}_{i2},\dots,p{}_{im}\}$, $p{}_{ij}\in\left\{\mathrm{0,1}\right\}$: Binary encoding, where 1 means the feature is selected, and 0 means the feature is not selected. In this case, $UB$ is the upper boundary for positions in feature space, $LB$ is the lower boundary for positions in feature space, and $T$ is the maximum number of iterations.

*Step 2: Fitness Function*.

The fitness function evaluates the quality of a feature subset:

6 $$Fitness\left(p{\text{}}_{i}\text{}\right)=\alpha\cdot{Accuracy}\left({X}_{p{\text{}}_{i}}\text{}\text{}\right)+\beta\cdot\left(1-\frac{\mid{X}_{p{\text{}}_{i}}\mid}{\left|X\right|}\right)$$

$Accuracy\left({X}_{p{}_{i}}\right)$ is the classification accuracy using a selected classifier trained on the feature subset ${X}_{p{}_{i}}$​​,$\left|{X}_{p{}_{i}}\right|$ is the number of selected features in $p{}_{i}$, and $\alpha,\beta$: are the weighting coefficients balancing accuracy and feature reduction.$\left|X\right|$ refers to the total number of original features in the complete feature set before any selection is applied.

*Step 3: Dynamic Exploration vs. Exploitation Balance*.

- Position Update: Grasshopper movement is guided by both attraction (exploitation) and repulsion (exploration):

7 $${p}_{i}^{(t+1)}=\sum_{j=1}^{N}{s}_{ij}.\frac{\left({p}_{j}-{p}_{i}\right)}{\parallel{p}_{j}-{p}_{i}\parallel}.r+g$$

${s}_{ij}$ is the social interaction strength, which dynamically adjusts exploration and exploitation.

8 $${s}_{ij}=c.\frac{{e}^{-d.\parallel{p}_{j}-{p}_{i}\parallel}}{\parallel{p}_{j}-{p}_{i}\parallel}$$

Where $c$ is the constant defining the influence magnitude, $d$is the decay factor controlling the balance, *r* is the randomness for exploration, reduced over iterations, and $g$ is the global best solution, encouraging convergence to optimal solutions.

- Dynamic Adjustment: Exploration and exploitation are balanced by adapting $c$ and $d$ dynamically over iterations:

9 $${c}_{t}={c}_{0}.\left(1-\frac{t}{T}\right)$$

10 $${d}_{t}={d}_{0}.\left(1-\frac{t}{T}\right)+\epsilon$$

${c}_{0}$, ${d}_{0}$ are initial values for interaction strength and decay factor, $t$ is the current iteration, $T$ is the total iterations, and $\epsilon$ is the small constant to maintain a minimum exploration level.

*Step 4: Selection and Elitism*.

- Evaluate all grasshopper solutions $P$ using the fitness function.
- Retain the top $k$ solutions (elitism) to ensure good features persist across iterations.

*Step 5: Stopping Criteria*.

The optimization stops when one of the following criteria is met:

- Maximum iterations $T$are reached.
- The fitness function shows no significant improvement over a predefined number of iterations.

*Step 6: Final Feature Subset*.

- The best-performing grasshopper solution is selected as the final feature subset ${X}_{s}$​.

The number of features selected after applying DGOA depends on several key factors. The initial feature set size ($m$) represents the total features extracted from the dataset. The configuration of the fitness function plays a crucial role, as it balances accuracy and feature reduction to determine how aggressively redundant features are pruned. DGOA dynamics also influence the process, where the exploration phase conducts a diverse search across the feature space to identify relevant features, and the exploitation phase fine-tunes the selection by focusing on subsets that maximize accuracy while minimizing redundancy. Additionally, stopping criteria, such as early stopping or convergence, impact the feature selection, balancing sufficient exploration and computational efficiency. Generally, DGOA significantly reduces the feature count to a fraction of the original, with the final count ($M$) varying based on algorithm settings. For instance, a dataset with $N=\mathrm{1,280}$ features is reduced to $M=100$or fewer, ensuring that critical biomarkers like microaneurysms, exudates, and hemorrhages are preserved while discarding irrelevant or noisy features.

In our case, the semantic clinical features, along with low-level visual patterns (e.g., edges, textures, color gradients), were not manually annotated, generated by a segmentation model, or sourced from a different dataset. Instead, they were derived from the same Kaggle EyePACS retinal fundus images used for model training. These features, though not explicitly labeled, contain rich visual information learned from hierarchical patterns within the retinal images. To semantically interpret these abstract features, multiple explainability tools were applied, revealing correlations between certain feature activations and clinically recognized signs of diabetic retinopathy—such as microaneurysm density, exudate presence, and hemorrhage spread. Attribution maps helped align feature responses with known lesion regions, enabling a post-hoc semantic mapping grounded in medical understanding, without relying on handcrafted features or explicit segmentation masks.

Although parameter adaptation strategies have been explored in previous metaheuristic optimizers, the proposed DGOA differs from the classical GOA through a task-oriented and coordinated dynamic control mechanism specifically designed for high-dimensional retinal feature selection. In standard GOA, the social interaction strength and comfort zone remain fixed throughout the optimization process, which often leads to premature convergence and limited search diversity when applied to complex medical imaging feature spaces. In contrast, DGOA introduces a multi-parameter dynamic adaptation framework in which the social interaction coefficient ($c$), decay factor ($d$), and stochastic exploration component ($r$) are simultaneously adjusted over iterations. This coordinated adaptation enables the algorithm to maintain a strong exploratory behavior during early iterations, allowing diverse feature subsets to be examined, while progressively shifting toward exploitation in later stages to refine compact and discriminative feature combinations. Such behavior is particularly critical in DR detection, where extracted deep features exhibit high redundancy and nonlinear interdependencies.

Table 2 summarizes the key distinctions between the classical GOA and the proposed DGOA, highlighting how dynamic parameter adaptation and task-specific design enable more robust and efficient feature selection for diabetic retinopathy detection. Table 3 summarizes the DGOA parameters used for feature selection, while Algorithm 1 presents the pseudocode outlining the feature selection process using DGOA.

**Table 2:** Conceptual comparison between standard GOA and proposed DGOA for feature selection in DR detection.

Aspect	Standard GOA	Proposed DGOA
Parameter Control	Static control parameters	Dynamic, iteration-dependent parameter adaptation
Exploration–Exploitation Balance	Fixed throughout optimization	Gradually transitions from exploration to exploitation
Suitability for High-Dimensional Features	Limited due to early convergence	Enhanced via adaptive search behavior
Feature Representation	Continuous, not task-specific	Binary encoding tailored for feature selection
Handling Feature Redundancy	Weak pruning capability	Progressive elimination of redundant features
Stability Across Runs	Sensitive to initialization	Improved robustness and convergence stability
Adaptation to Medical Imaging	Generic optimization behavior	Domain-driven design for retinal fundus features
Risk of Local Optima	Higher	Reduced through dynamic social interaction control

**Table 3:** DGOA parameter configuration for feature selection.

Parameter	Symbol	Value/Range	Description
Population size	$N$	30	Number of grasshoppers (candidate feature subsets)
Feature dimension	$m$	Dataset-dependent	Total number of extracted features
Maximum iterations	$T$	100	Maximum optimization iterations
Global control coefficient (initial)	${C}_{max}$	1.0	Controls initial exploration strength
Global control coefficient (final)	${c}_{min}$​	0.1	Encourages exploitation near convergence
Initial decay factor	${d}_{0}$	1.0	Distance-based interaction decay
Interaction strength (initial)	${C}_{0}$	1.0	Initial social interaction magnitude
Minimum decay offset	$\epsilon$	0.01	Prevents complete loss of exploration
Randomness factor	$r$	[0, 1]	Reduced linearly over iterations
Fitness weight (accuracy)	$\alpha$	0.7	Importance of classification accuracy
Fitness weight (reduction)	$\beta$	0.3	Importance of feature reduction
Elitism size	$k$	2	Number of top solutions preserved
Early stopping patience	—	10 iterations	Stop if no improvement
Encoding	—	Binary (0/1)	Feature selected or not
Attractive length scale	$l$	1.5	Controls interaction range between grasshoppers
Attraction intensity	$f$	0.5	Strength of attraction in social force function
Gravity term	$G$	Global best solution	Pulls population toward best solution
Wind advection	$W$	Zero vector	Disabled to avoid directional bias
Transfer function	—	Sigmoid	Continuous-to-binary mapping

To ensure reproducibility and stable optimization behavior, the DGOA-based feature selection experiments were conducted over 10 independent runs, a standard practice in metaheuristic optimization to account for stochastic variability, with a fixed random seed of 42 applied to initialize population positions. A random seed is used to initialize the positions of the grasshopper population in a reproducible way, ensuring that stochastic variations in DGOA runs can be consistently replicated. The seed value of 42 was chosen arbitrarily, following a common convention in computational experiments as a fixed, reproducible number^17,18^. The key hyperparameters used for DGOA are listed in Table 3. Specifically, a population size of 30 was chosen to provide sufficient search diversity while maintaining computational feasibility, and a maximum iteration limit of 100 ensures convergence without premature stagnation. The initial global attraction coefficient ${C}_{max}=1.0$ encourages exploration in the early stages of optimization, while the final attraction coefficient ${c}_{min}=0.1$ promotes exploitation and convergence as the algorithm progresses. Dynamic adaptation is enabled to allow a smooth transition from exploration to exploitation. These settings were selected to balance search efficiency, solution quality, and computational cost, resulting in robust and reproducible feature selection outcomes across multiple runs.

**Algorithm 1:**
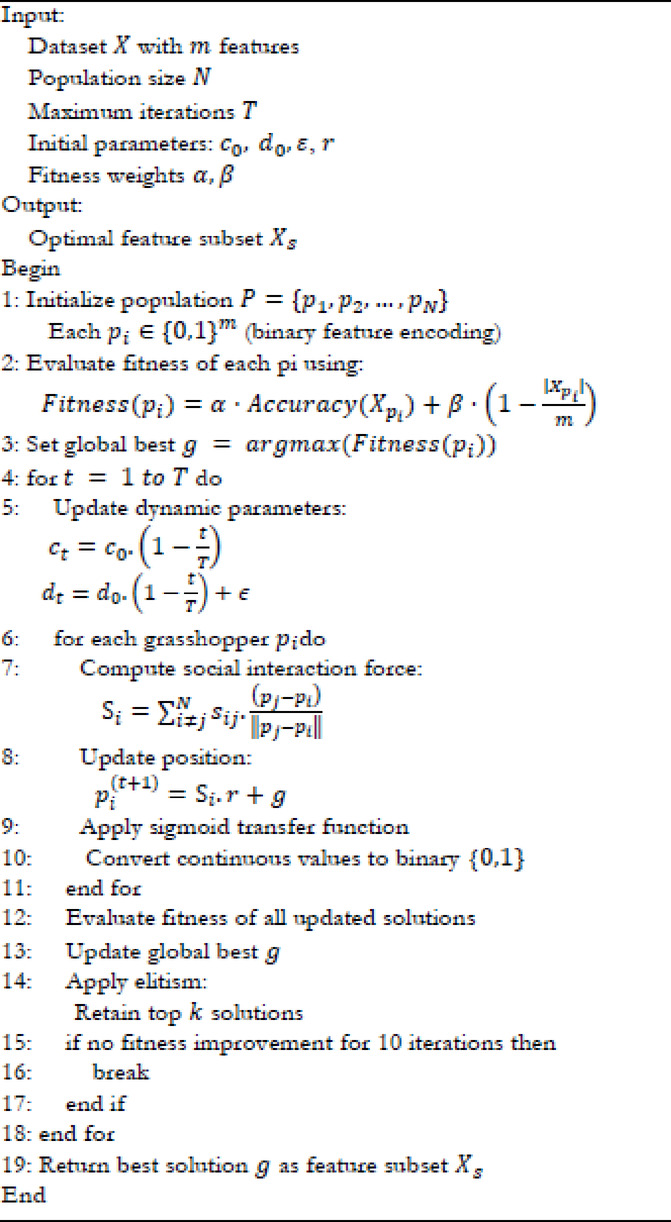
DGOA for feature selection.

### Ensemble learning classifier

Ensemble learning classifiers play a significant role in DR detection in retinal fundus images by combining the predictions of multiple models to improve the overall performance, robustness, and reliability of the detection system^56^. Ensemble learning can mitigate the weaknesses of individual models, enhance generalization, and better handle complex patterns in retinal fundus images that indicate DR^57^. Ensemble learning enhances DR detection by combining weak learners to generate stronger, more accurate predictions. Methods like bagging, boosting, and stacking aggregate outputs from individual classifiers, enabling models such as RFs (bagging) and Gradient Boosting to detect subtle DR features. These techniques address class imbalance in retinal fundus datasets by giving higher weights to misclassified instances, improving focus on minority classes and varying DR severity stages^58^.

According to the literature, boosting methods, particularly Gradient Boosting and its advanced variants like XGBoost and LightGBM, often outperform bagging and stacking for DR detection due to their ability to handle imbalanced datasets and focus on hard-to-classify instances^58^. Boosting assigns higher weights to misclassified samples, enabling the model to better detect subtle signs of DR across different severity levels. While bagging methods like RFs are effective at reducing overfitting and improving generalization, they may not perform as well when the dataset is imbalanced. Stacking, though promising for combining diverse models, can be computationally intensive and less interpretable, making boosting the preferred choice for its balance of accuracy, efficiency, and handling of imbalanced data in DR detection tasks^58^.

For DR detection, LightGBM is often preferred over XGBoost due to its superior computational efficiency and scalability when dealing with large datasets and high-dimensional features^59^. LightGBM employs a leaf-wise tree growth strategy instead of the level-wise approach used in XGBoost, enabling it to achieve better accuracy with fewer iterations by focusing on reducing loss more effectively at each step. Additionally, LightGBM supports histogram-based learning, which reduces memory usage and speeds up training, making it more suitable for resource-constrained environments or when working with high-resolution retinal fundus images. While XGBoost is highly robust and performs well, LightGBM’s faster training speed and efficiency, coupled with its capability to handle class imbalances through customizable loss functions and hyperparameter tuning, make it a compelling choice for DR detection tasks^59,60^. Below is the step-by-step explanation for an ensemble deep learning model integrating base classifiers Support Vector Machine (SVM), Bayesian Network (BN), and Decision Tree (DT) using LightGBM.

*Step 1: Base Classifier Training*.

Train individual classifiers (SVM, BN, and DT) on extracted feature vectors to predict the presence of DR^61,62^.

- SVM: given the feature vectors ${X}_{features}$.

11 $$decisionfunctionf\left(x\right)=sign\left(w.x+b\right)$$

12 $$min\frac{1}{2}{\parallel{w}\parallel}^{2}+\lambda\sum_{I=1}^{n}{\xi}_{i}$$

Optimization problem

13 $$\mathrm{S}\mathrm{u}\mathrm{b}\mathrm{j}\mathrm{e}\mathrm{c}\mathrm{t}\mathrm{t}\mathrm{o}{y}_{i}\left(w.{x}_{i}+b\right)\ge1-{\xi}_{i}{\xi}_{i}\ge0$$

$w$ is the weight vector that defines the orientation of the separating hyperplane in the feature space, $b$ is the bias term (offset) that determines the hyperplane’s position relative to the origin, $\lambda$ is the regularization parameter, which balances the trade-off between maximizing the margin and minimizing the classification error. A higher $\lambda$ puts more emphasis on reducing classification errors but may lead to overfitting, and ${\xi}_{i}$ is the slack variable for the *i*-th data point, representing the amount by which the data point violates the margin ${\xi}_{i}\ge0$. The output is the class probabilities (${p}_{SVM}$​).

- BN: given the feature vectors ${X}_{features}$. Bayesian inference calculates the posterior probability for each class:

14 $$P\left({C}_{k}|X\right)=\frac{P\left(X|{C}_{k}\right).P\left({C}_{k}\right)}{P\left(X\right)}$$

$P\left({C}_{k}|X\right)$ is the posterior probability of class ${C}_{k}$​ given features $X$, $P\left(X|{C}_{k}\right)$ is the likelihood of $X$ given class ${C}_{k}$​, $.P\left({C}_{k}\right)$ is the prior probability of class ${C}_{k}$​, and $P\left(X\right)$ is the evidence. The output is the class probabilities (${p}_{BN}$​).

- DT: Constructs a tree by recursively partitioning data based on feature thresholds.

15 $$SplitCriterion:G=\sum_{i=1}^{m}\frac{{n}_{i}}{N}H\left(i\right)$$

$G$ is the information gain, ${n}_{i}$ is the number of samples in node $i$, $N$ is the total samples, and $H\left(i\right)$ is the entropy or Gini impurity at node $i$. The output is the class probabilities (${p}_{DT}$​).

The selection of SVM, BN, and DT as base classifiers is motivated by their complementary learning mechanisms and inductive biases when operating on high-dimensional retinal feature representations. SVM is margin-based and excels at separating complex decision boundaries in high-dimensional spaces, BN captures probabilistic dependencies and uncertainty among features, and DT provides rule-based, hierarchical partitioning that is particularly effective for modeling non-linear feature interactions. These fundamentally different modeling principles enable each classifier to focus on distinct aspects of the feature space, increasing diversity within the ensemble.

*Step 2: Ensemble with LightGBM*.

Given the predictions from Base Classifiers$\left({p}_{SVM},{p}_{BN},{p}_{DT}\right)$. LightGBM builds decision trees sequentially, where each new tree corrects the errors made by the previous ones, and uses a unique leaf-wise growth strategy to maximize efficiency and accuracy. By optimizing a specified loss function (e.g., binary cross-entropy for classification), LightGBM splits nodes based on the highest information gain, leading to faster convergence and better handling of large datasets with high-dimensional features^59^.

- 2.1: Objective Function: For binary classification (e.g., DR vs. No DR):

16 $$ObjectiveFunction=-\frac{1}{N}\sum_{i=1}^{N}\left[{y}_{i}.log\left({p}_{i}\right)+\left(1-{p}_{i}\right).log\left(1-{p}_{i}\right)\right]$$

${y}_{i}$ is the ground truth label (1 for DR, 0 for No DR), and ${p}_{i}$is the predicted probability for sample $i$.

For multi-class classification with $K$ classes (e.g.,$K=5$), the objective function for LightGBM is based on the softmax function and the categorical cross-entropy loss.

17 $$ObjectiveFunction=-\frac{1}{N}\sum_{i=1}^{N}\sum_{k=1}^{K}{y}_{i,k}.log\left({p}_{i.k}\right)$$

${y}_{i,k}$ is the binary indicator (1 if sample $i$ belongs to class $k$, otherwise 0), and ${p}_{i.k}$ is the predicted probability for sample $i$ and class $k$, calculated using the softmax function.

18 $${p}_{i.k}=\frac{exp\left({z}_{i,k}\right)}{\sum_{j=1}^{K}exp\left({z}_{i,j}\right)}$$

Here ${z}_{i,k}$​ is the raw score (logit) for class $k$ from the model output for sample$i$.

- 2.2: Gradient and Hessian:

Gradient ($g$): First derivative of the loss.

19 $${g}_{i}=\frac{\partial{L}}{\partial{p}_{i}}={p}_{i}-{y}_{i}$$

$L$ is the loss function, which quantifies the error between the predicted and true values.

For binary classification, it is typically the binary cross-entropy loss as in Eq. (16)

Hessian ($h$): Second derivative of the loss.

20 $${h}_{i}=\frac{{\partial}^{2}L}{\partial{{p}_{i}}^{2}}={p}_{i}\left(1-{p}_{i}\right)$$

- 2.3: Leaf-Wise Tree Growth:

For each split, select the feature $j$ that maximizes gain:

21 $${G}_{split}=\frac{{\left({\sum}_{i\in{L}}{g}_{i}\right)}^{2}}{{\sum}_{i\in{L}}{h}_{i}+\lambda}+\frac{{\left({\sum}_{i\in{R}}{g}_{i}\right)}^{2}}{{\sum}_{i\in{R}}{h}_{i}+\lambda}-\frac{{\left({\sum}_{i\in{L}\cup{R}}{g}_{i}\right)}^{2}}{{\sum}_{i\in{L}\cup{R}}{h}_{i}+\lambda}$$

$L,R$ are the left and right child nodes, and $\lambda$ is the regularization term. Herein, $\lambda$ controls the complexity of the model by penalizing overly large weights in the decision trees, which helps reduce overfitting. The ideal value depends on the dataset and task; small $\lambda$ values (e.g., 0.1) allow the model to fit more complex patterns, while larger values (e.g., 10) enforce stronger regularization, encouraging simpler models. The best $\lambda$ is usually selected based on cross-validation performance, balancing the trade-off between underfitting and overfitting.

- 2.4: Update Predictions:

Combine base predictions with LightGBM:

22 $${\widehat{y}}_{new}={\widehat{y}}_{old}+\eta.TreeOutput$$

$\eta$ is the learning rate, which scales the contribution of the new tree’s output to prevent overfitting and ensure gradual improvement. Smaller values (e.g., 0.01to 0.10) lead to slower but more stable training, while larger values may risk overshooting optimal solutions. $TreeOutput$ is the output of the new decision tree, representing the correction or adjustment the tree makes to reduce the current residual error. This is calculated by minimizing the loss function for the residuals and serves as the model’s response to the remaining error in predictions. The output is the final prediction probabilities ($\widehat{y}$).

- 2.5: Thresholding and Classification.

Use a threshold $T$ (e.g.,$T=0.5$) to classify DR vs. No DR:

23 $$Class=\left\{\begin{array}{cc}NoRD&if\widehat{y}<T\\DR&if\widehat{y}\ge{T}\end{array}\right.$$

For a 5-class classification problem with the classes “No DR,” “Mild DR,” “Moderate DR,” “Severe DR,” and “Proliferative DR,” the decision rule can be modified as follows:

24 $$Class=\left\{\begin{array}{cc}\text{}NoDR&if\widehat{y}<{T}_{1}\\MildDR&if{T}_{1}\le\widehat{y}<{T}_{2}\\\begin{array}{c}ModerateDR\\\begin{array}{c}SevereDR\\ProliferativeDR\end{array}\end{array}&\begin{array}{c}if{T}_{2}\le\widehat{y}<{T}_{3}\\\begin{array}{c}if{T}_{3}\le\widehat{y}<{T}_{4}\\\widehat{y}\ge{T}_{4}\end{array}\end{array}\end{array}\right.$$

${T}_{1}{T}_{2},{T}_{3},and{T}_{4}$ are predefined thresholds that partition the prediction space into the 5 classes. These thresholds were determined using a data-driven optimization strategy based on validation-set performance. Specifically, the predicted probability outputs of the LightGBM ensemble were analyzed on a held-out validation subset to identify optimal class boundaries. The thresholds were selected by maximizing the macro-averaged F1-score while preserving the clinically meaningful ordinal progression of DR severity.

To achieve this, class-wise probability distributions were examined, and adjacent thresholds were positioned at the intersection points where misclassification between neighboring DR stages was minimized. This approach ensures that early-stage DR cases (e.g., Mild and Moderate) are not overshadowed by dominant classes, which is critical in imbalanced retinal datasets. The final threshold values were fixed as ${T}_{1}=0.20$, ${T}_{2}=0.4$, ${T}_{3}=0.65$, and ${T4}_{4}=0.85$, reflecting increasing confidence requirements for more severe DR stages. This validation-guided thresholding strategy improves sensitivity for early DR detection while maintaining high specificity for advanced stages, aligning the model’s decision boundaries with both statistical performance and clinical diagnostic practice.

Rather than acting as an additional standalone classifier, LightGBM is employed as a meta-learner that aggregates the probabilistic outputs of SVM, BN, and DT. By learning from these prediction-level features, LightGBM adaptively assigns higher importance to the most reliable classifier for a given input pattern. Its leaf-wise growth strategy enables efficient learning of nonlinear relationships between base predictions, leading to improved robustness and generalization without excessive computational overhead. This stacked ensemble design therefore balances complexity and performance, achieving superior accuracy compared to any single classifier or naïve ensemble.

LightGBM was chosen over XGBoost because it grows trees leaf-wise, expanding the leaf with the largest loss reduction, whereas XGBoost grows trees level-wise, expanding all nodes at the same depth before moving deeper. This leaf-wise growth allows LightGBM to achieve faster convergence and higher accuracy with fewer trees, making it more efficient for large datasets.

In the proposed stacked ensemble framework, the outputs from the base classifiers are used directly as probability estimates for LightGBM without additional calibration. These default probabilities are sufficiently well-distributed to represent confidence levels in DR predictions. Preliminary experiments comparing calibrated and uncalibrated probabilities (using Platt scaling) showed negligible differences in meta-learner performance (< 0.5% change in F1-score). By using default probability estimates, the ensemble maintains computational efficiency, avoids unnecessary complexity, and preserves interpretability of the base classifier outputs, while still enabling LightGBM to effectively learn patterns in the prediction-level feature space.

To comprehensively evaluate the proposed DGOA-ensemble model, a series of experiments were designed to assess different aspects of its performance, including accuracy, robustness, generalization, computational efficiency, and interpretability. The experimental setups involve comparing the model against baseline feature selection and classification methods, conducting ablation studies to quantify the impact of DGOA-based feature selection, testing generalization across multiple dataset splits and cross-validation schemes, and evaluating robustness under noisy and augmented retinal fundus images. Additional experiments focus on handling class imbalance to improve early-stage DR detection, assessing statistical significance to ensure reliability of observed improvements, and performing explainability analysis using SHAP and LIME to interpret feature importance. For fair comparison, all experiments employ consistent dataset partitions, hyperparameters, and evaluation conditions across different configurations and competing methods.

The evaluation metrics were carefully selected to capture both classification performance and practical applicability in clinical settings. Key metrics include accuracy, precision, recall, F1-score, and AUC-ROC to assess predictive effectiveness, while training and inference times, as well as memory usage, are reported to evaluate computational efficiency and scalability. For minority class detection, especially mild DR, metrics such as Precision-Recall curves and class-specific F1-scores are used to ensure balanced performance. Statistical significance tests, including the Wilcoxon Signed-Rank Test with confidence intervals, validate that observed improvements are consistent and reproducible. The detailed mapping of experiments to their corresponding metrics is summarized in Table 2, providing a clear framework for interpreting subsequent results.

To ensure the reproducibility of the proposed approach, all experiments were conducted using a specified computational environment, including hardware and software configurations. The experiment was carried out on a system equipped with an Intel(R) Core(TM) i7 processor and 8 GB of RAM. This hardware configuration, though modest, provided sufficient computational power to support deep learning computations and analysis tasks effectively. This setup employs Python 3.8 for compatibility with current libraries and uses Anaconda for package management. The proposed DR detection pipeline uses multiple Python libraries, each serving a specific purpose. Pandas and NumPy handle data preprocessing and feature organization, while scikit-learn supports standard machine learning utilities such as train-test splitting, feature standardization, and training base classifiers (SVM, Bayesian Network, and Decision Tree). TensorFlow/Keras is employed for implementing and training any deep learning components within the ensemble when required. The LightGBM library serves as the meta-learner in the stacked ensemble, aggregating the probabilistic outputs of the base classifiers, and NiaPy is used to optimize LightGBM’s hyperparameters efficiently without introducing additional ensemble layers. Finally, Matplotlib and Seaborn are used for visualization of model performance, including accuracy, precision, and ROC curves.

With this computational setup in place, we conducted extensive evaluations to ensure the model’s robustness and generalizability across different data partitions and validation strategies. Generalization refers to how well the model performs on unseen data, ensuring that it does not overfit to a specific training set. To achieve this, multiple train-test splits (80 − 20, 70 − 30, and 60 − 40) are used to analyze how varying the amount of training data impacts performance. Additionally, k-fold cross-validation (5-fold and 10-fold) is implemented to ensure robust evaluation by training the model on different subsets of the data, reducing the dependency on any single partition.

## Experimental results and discussions

### Datasets, evaluation metrics, and implementation

In this section, the proposed model is validated using the APTOS 2019 Blindness Detection dataset, while the Kaggle EyePACS dataset is employed for model training and initial evaluation, as described in the data acquisition section. The APTOS 2019 dataset is publicly available on Kaggle and is widely used for DR analysis. In our experiments, the dataset was accessed through a user-curated Kaggle repository (https://www.kaggle.com/datasets/kssanjaynithish03/retinal-fundus-images), which mirrors the original APTOS 2019 challenge data. This repository contains the same retinal fundus images and diagnostic labels as the official release, with differences limited to file organization and preprocessing format. The dataset comprises thousands of high-quality retinal fundus images captured using specialized fundus cameras and stored in JPEG format, with file sizes ranging from approximately 500 KB to several megabytes, depending on image resolution and quality. The use of APTOS 2019 as an independent validation dataset enables a rigorous assessment of the generalization performance of the proposed model across different data distributions.

The images were acquired under varying illumination conditions, focus levels, and retinal orientations, which enhances model robustness in real-world screening scenarios. Each image is annotated according to clinically recognized DR severity levels, ranging from no apparent signs to severe proliferative diabetic retinopathy, enabling the model to learn from a wide spectrum of disease manifestations. The dataset also includes images exhibiting additional retinal abnormalities, contributing to more comprehensive feature learning. Furthermore, the data were collected from diverse patient populations, capturing variations in age, ethnicity, and underlying health conditions, which supports model generalization across different demographics. The availability of high-resolution images facilitates the extraction of fine-grained retinal features such as microaneurysms, hemorrhages, exudates, and neovascularization, all of which are critical indicators for accurate DR diagnosis.

To comprehensively evaluate the effectiveness and robustness of the proposed DGOA-ensemble model, a series of experiments were conducted, each designed to assess different aspects of its performance. These experiments aim to compare the model with existing methods, analyze its contribution to feature selection, and evaluate its generalization ability across different dataset splits. Additionally, robustness tests were performed to examine the model’s response to noisy data and augmentation, while computational efficiency was assessed in terms of scalability and speed. Further analysis was conducted to measure the model’s handling of class imbalance, ensuring fair performance across minority classes. To validate the reliability of improvements, statistical significance tests were carried out, and an explainability analysis was performed using SHapley Additive Explanations (SHAP) and Local Interpretable Model-agnostic Explanations (LIME) visualizations to understand the impact of selected features. The key objectives and evaluation metrics for each experiment are summarized in Table 4.

**Table 4:** Summary of key experiments.

Experiment	Objective	Key metrics
Baseline Comparison	Compare DGOA-ensemble with existing traditional optimization and classification methods	Accuracy, F1-score, AUC-ROC, inference time
Ablation Study	Assess DGOA’s impact on feature selection	Accuracy change, feature reduction, training time
Dataset Split Testing	Evaluate generalization ability	Cross-validation results, standard deviation of accuracy
Noisy Data & Augmentation	Test robustness against real-world distortions	Accuracy drop, robustness factor
Computational Efficiency	Measure scalability and speed	Training time, inference speed, memory usage
Class Imbalance Handling	Evaluate performance on minority classes	AUC-ROC, Precision-Recall, F1-score
Statistical Significance	Confirm reliability of improvements	*p*-value, confidence intervals
Explainability Analysis	Understand feature importance	SHAP/LIME visualizations, feature impact scores
Comparative Analysis	Compare the suggested model with state-of-the art diabetic retinopathy detection approaches	Accuracy, F1-score, AUC-ROC

To ensure full reproducibility of the proposed DGOA-ensemble framework, all key hyperparameters for DGOA and the employed classifiers (SVM, DT, BN, and LightGBM) are explicitly reported in Table 5, along with their justification. These parameters were selected based on commonly accepted defaults in the literature or tuned through preliminary validation experiments to balance performance, stability, and computational efficiency^63–65^. These configurations were fixed across all experiments to guarantee fair comparisons and stable performance evaluation. Differences in reported metrics reflect different experimental goals, not inconsistencies or errors. Minor variations are expected due to dataset size, split strategy, and evaluation protocol.

**Table 5:** Key hyperparameters used for reproducibility.

Component	Hyperparameter	Value	Justification
DGOA	Population size	30	Provides sufficient search diversity while keeping computational cost manageable
	Maximum iterations	100	Ensures convergence of feature selection without premature stagnation
	Initial attraction coefficient $({c}_{max}$)	1.0	Encourages global exploration at early stages
	Final attraction coefficient (${c}_{min}$)	0.1	Promotes exploitation and convergence in later iterations
	Dynamic adaptation	Enabled	Allows smooth transition from exploration to exploitation
SVM	Kernel	RBF	Captures nonlinear relationships in retinal features
	Regularization parameter (C)	1.0	Balances margin maximization and misclassification penalty
	Kernel width (γ)	1/number of features	Standard heuristic for stable decision boundaries
	Multiclass strategy	One-vs-One	Commonly used for multi-class medical classification tasks
DT	Split criterion	Gini impurity	Efficient impurity measure for classification
	Maximum depth	None (unrestricted)	Allows full feature interaction after DGOA reduction
	Minimum samples per split	2	Default setting ensuring sufficient node purity
BN	Structure learning	Naïve Bayes assumption	Reduces complexity and avoids overfitting in high-dimensional data
	Parameter estimation	Maximum Likelihood	Standard probabilistic estimation method
LightGBM	Number of boosting iterations	100	Balances performance and training time
	Learning rate (η)	0.05	Ensures stable convergence and prevents overfitting
	Maximum tree depth	−1 (leaf-wise growth)	Enables adaptive tree complexity
	Number of leaves	31	Default value ensuring model expressiveness
	Objective function	Multiclass cross-entropy	Suitable for multi-stage DR classification
	Class weighting	Enabled	Mitigates class imbalance across DR severity levels

### Experiment 1: performance comparison with baseline methods

The primary objective of this experiment is to evaluate the performance of the proposed DGOA-based ensemble model in comparison with traditional optimization and classification methods. Traditional feature selection techniques, including GA, PSO, and standard GOA, are compared against the DGOA approach. The classification models used for evaluation include SVM, Random Forest (RF), and a simple CNN, all trained without ensemble learning. The baseline CNN consists of three convolutional layers with ReLU activations, each followed by max-pooling, and two fully connected layers for classification, representing a lightweight, standard architecture. This is distinct from the EfficientNet-B0 backbone used for feature extraction in the proposed DGOA-based ensemble, ensuring that the baseline comparison reflects conventional non-ensemble models. The models are trained and tested using two different train-test splits (80–20 and 70–30). Performance is evaluated using accuracy, precision, recall, and F1-score, while AUC-ROC curves measure model discrimination capability. Additionally, training and inference times are recorded to assess computational efficiency.

**Table 6:** Comparative performance of DGOA-ensemble model against traditional optimization and classification methods.

Feature selection	Classifier	Accuracy (%)	Precision	Recall	F1-score	AUC-ROC	Training time (s)	Inference time (s)
GA	SVM	85.2	0.84	0.82	0.83	0.88	120	1.5
	RF	86.5	0.85	0.84	0.84	0.89	150	1.8
	CNN	89.1	0.88	0.87	0.87	0.92	200	2.5
PSO	SVM	83.8	0.82	0.81	0.81	0.86	130	1.6
	RF	85.0	0.84	0.83	0.83	0.88	160	1.9
	CNN	87.9	0.87	0.86	0.86	0.91	210	2.7
GOA	SVM	86.0	0.85	0.83	0.84	0.89	125	1.4
	RF	88.2	0.87	0.86	0.86	0.91	170	2.0
	CNN	90.3	0.89	0.88	0.89	0.93	220	2.8
DGOA (Proposed)	Ensemble	**94.1**	**0.93**	**0.92**	**0.93**	**0.97**	**190**	**1.9**

The results shown in Table 6 indicate that the proposed DGOA-ensemble model outperforms traditional optimization and classification approaches across all performance metrics. With an accuracy of 94.1% and an AUC-ROC score of 0.97, the model demonstrates superior discriminatory power. Additionally, its F1-score of 0.93 suggests a well-balanced classification performance, effectively handling both false positives and false negatives. The efficiency of the model is also notable, as it achieves the highest performance while maintaining a lower inference time (1.9s) than standalone CNN models. Compared to traditional feature selection methods, GOA performs better than GA and PSO in most cases, highlighting its effectiveness in selecting relevant features. The integration of ensemble learning in DGOA further enhances classification accuracy and robustness. Despite slightly higher training time (190s) compared to other models, the proposed approach remains computationally feasible for real-world applications, making it a highly promising solution for automated DR detection.

The DGOA achieves superior feature selection results compared to traditional GOA due to its dynamic adaptation mechanism, which enhances exploration and exploitation during the optimization process. Unlike standard GOA, which relies on a fixed search strategy, DGOA dynamically adjusts the movement patterns of grasshoppers based on convergence trends, preventing premature stagnation in local optima. This adaptability enables more effective identification of the most relevant and discriminative features, leading to improved classification accuracy and robustness. Additionally, DGOA integrates an ensemble learning approach that leverages the strengths of multiple classifiers, further refining feature selection and boosting overall model performance.

### Experiment 2: ablation study on the effect of dgoa in feature selection

The objective of this ablation study is to systematically quantify the individual contributions **of** DGOA-based feature selection and ensemble learning **w**ithin the proposed framework. To this end, three controlled experimental settings are evaluated: (i) a baseline model trained on all raw EfficientNet-B0 features without feature selection, (ii) a model using DGOA-selected features with a single LightGBM classifier, and (iii) the full proposed DGOA-based ensemble model combining SVM, Bayesian Network (BN), Decision Tree (DT), and LightGBM. All models are trained using the same dataset, identical hyperparameters, and consistent evaluation metrics to ensure a fair comparison. All models use the same dataset, classification algorithm, hyperparameters, and evaluation metrics to ensure a fair comparison. The number of selected features before and after DGOA optimization is recorded to measure dimensionality reduction. Performance metrics such as accuracy, precision, and F1-score are compared to determine if the optimized feature subset improves classification capability. Additionally, computation time for both feature selection and training is measured to assess the efficiency trade-offs introduced by DGOA.

**Table 7:** Ablation study on the effect of DGOA-based feature selection and ensemble learning.

Experiment setup	Classifier configuration	Features selected	Accuracy (%)	Precision	F1-Score	Feature selection time (s)	Training time (s)
Without Feature Selection	LightGBM	All Features (*N* = 1,280)	85.3	0.82	0.81	–	120
DGOA-Based Feature Selection	LightGBM	Reduced (M = 100, M < < N)	91.2	0.89	0.88	5.1	88
**Proposed Full Model**	**DGOA + Ensemble (SVM + BN + DT + LightGBM)**	**Reduced (M = 100)**	**94.3**	**0.92**	**0.91**	**5.1**	**85**

As reported in Table 7, applying DGOA-based feature selection alone (DGOA + LightGBM) improves accuracy from 85.3% to 91.2%, demonstrating the effectiveness of DGOA in removing redundant and noisy features while preserving discriminative information. Notably, incorporating ensemble learning on top of DGOA-selected features further increases accuracy to 94.3%, with corresponding improvements in precision (0.92) and F1-score (0.91). This confirms that while DGOA substantially enhances performance through optimized feature selection, the ensemble strategy provides an additional performance gain by utilizing complementary decision boundaries from heterogeneous classifiers. The combined DGOA–ensemble framework therefore achieves superior robustness, generalization, and classification accuracy compared to using either feature selection or a single classifier alone.

Although ensemble models generally require higher computational cost due to the involvement of multiple classifiers, the proposed DGOA-based ensemble demonstrates a slightly lower training time (85s) compared to the single-classifier DGOA + LightGBM configuration (88s), corresponding to a 3.4% reduction in training time. This behavior can be attributed to the highly compact feature subset produced by DGOA (M = 100 features), which significantly reduces model complexity and accelerates convergence for all ensemble members. In particular, individual components such as SVM, Bayesian Network, and shallow Decision Trees converge rapidly in a low-dimensional feature space and do not require extensive iterative optimization. In contrast, LightGBM alone relies on sequential gradient boosting, which involves iterative tree construction and can incur higher training overhead even with reduced features. Consequently, the complementary learning dynamics and efficient convergence of the heterogeneous ensemble offset the expected computational overhead, resulting in faster overall training despite the use of multiple classifiers. These results confirm that the high performance of the proposed model is not solely due to EfficientNet-B0 feature extraction, but is primarily driven by the adaptive and robust feature selection enabled by DGOA, which enhances model generalization and classification capability.

Furthermore, Table 8 presents a comparative analysis between the classical GOA and the proposed DGOA in the context of feature selection for DR detection. To ensure a fair and controlled evaluation, both algorithms were tested using the same classifier with identical hyperparameter settings and experimental configuration. The objective of this comparison is to evaluate the effectiveness of the dynamic parameter adaptation strategy in DGOA, specifically in terms of the number of selected features, classification performance, convergence speed, and solution stability. By directly comparing the static parameter configuration of GOA with the multi-parameter dynamic adaptation of DGOA under identical conditions, this analysis highlights how algorithmic modifications influence both the quality and compactness of the selected feature subset, which is critical for improving downstream classification accuracy in high-dimensional retinal image datasets.

**Table 8:** Comparison between standard GOA and proposed DGOA for feature selection in DR detection.

Method	Control strategy	Avg. selected features	Accuracy (%)	Sensitivity (%)	Specificity (%)	Convergence speed	Stability (Std. Dev.)
GOA	Static parameters	180 ± 12	90.3	88.5	91.5	Slow	High variance
DGOA (Proposed)	Dynamic multi-parameter adaptation	94 ± 8	94.1	92.3	95.0	Faster	Lower variance

As shown in Table 8, the proposed DGOA selects a significantly smaller subset of features, 94 ± 8 features, compared to 180 ± 12 features in standard GOA, indicating a more compact and efficient representation of discriminative retinal characteristics. In terms of classification performance, DGOA achieves an accuracy of 94.1%, surpassing the 90.3% achieved by standard GOA. This improvement is accompanied by higher sensitivity (92.3% vs. 88.5%) and specificity (95.0% vs. 91.5%), demonstrating that DGOA not only reduces redundancy but also preserves clinically relevant features critical for detecting microaneurysms, hemorrhages, and exudates. Additionally, DGOA exhibits faster convergence and lower variance across multiple runs, reflecting greater stability and robustness in the feature selection process. Collectively, these results justify the superiority of the proposed DGOA, as its dynamic adaptation mechanism effectively balances exploration and exploitation, leading to a more compact, accurate, and reliable feature subset for DR detection compared to the classical GOA.

### Experiment 3: generalization across different dataset splits

The objective of this experiment is to evaluate the model’s ability to generalize across different dataset partitions when applied to Retinal Fundus Image datasets. Key performance metrics include the standard deviation of accuracy across different splits to measure consistency, model variance over repeated training sessions to assess stability, and cross-validation results to determine robustness. These evaluations provide insights into the model’s reliability in detecting retinal abnormalities across diverse data distributions. Several publicly available Retinal Fundus Image datasets are used in this experiment, each with unique characteristics. The DIARETDB1 (https://paperswithcode.com/dataset/diaretdb1) dataset consists of 89 images, including both normal and diabetic retinopathy cases, with expert-annotated lesions such as microaneurysms and hemorrhages. This dataset is useful for early-stage diabetic retinopathy detection. The MESSIDOR (https://paperswithcode.com/dataset/messidor-1) dataset, which contains 1,200 retinal images, provides detailed diabetic retinopathy grading, making it suitable for assessing the severity of the disease. Another significant dataset is the DRIVE (https://www.kaggle.com/datasets/zhz638/drive-dataset) (Digital Retinal Images for Vessel Extraction) dataset, which includes 40 fundus images with manually segmented blood vessels, commonly used for evaluating vessel segmentation models. Additionally, the APTOS 2019 Blindness Detection (https://www.kaggle.com/competitions/aptos2019-blindness-detection) dataset comprises over 3,600 fundus images labeled across five severity levels of diabetic retinopathy, allowing multi-class classification experiments. By applying the generalization analysis to these datasets, we can assess how well the model adapts to variations in image quality, disease severity, and labeling consistency, ultimately improving its reliability in real-world clinical applications.

Each dataset used in this study was treated as an independent experimental setting. The proposed model was trained and evaluated separately on each dataset to account for differences in dataset size, labeling strategy, and clinical focus. The Retinal Fundus Images dataset from Kaggle and the APTOS 2019 dataset were used for diabetic retinopathy classification tasks, with APTOS 2019 supporting multi-class severity grading. DIARETDB1 was employed for early-stage diabetic retinopathy detection, while MESSIDOR was used to evaluate disease severity grading performance. The DRIVE dataset was included to assess the model’s ability to generalize to vessel-related retinal structures. No dataset was merged or reused for cross-dataset training, ensuring that performance results reflect dataset-specific generalization rather than transfer learning effects.

For each dataset, all available images were used and partitioned according to three train–test split configurations: 80–20, 70–30, and 60–40. These splits were applied randomly while preserving class distribution where applicable. Smaller datasets such as DIARETDB1 and DRIVE naturally resulted in fewer training samples, which explains the higher variance observed in their performance metrics. Larger datasets, including MESSIDOR and APTOS 2019, provided more stable training and lower variability across splits.

The proposed model was initialized with the same architectural configuration and hyperparameters for all datasets to ensure consistency. However, the model was re-trained from scratch for each dataset and each train–test split. No weight sharing or fine-tuning across datasets was performed. This approach ensures that the reported results reflect the model’s ability to learn dataset-specific features rather than benefiting from prior exposure to similar data.

In addition to fixed train–test splits, 5-fold and 10-fold cross-validation were conducted independently for each dataset. In k-fold cross-validation, the dataset was randomly divided into k equally sized folds. During each iteration, one fold was used for testing while the remaining folds were used for training. The final reported cross-validation accuracy represents the average performance across all folds. This evaluation strategy minimizes bias due to data partitioning and provides a robust estimate of model generalization.

Model performance was evaluated primarily using classification accuracy. To assess stability, the standard deviation and variance of accuracy were computed across multiple training runs with different random initializations. These metrics provide insights into the sensitivity of the model to dataset size and partitioning, particularly in small and imbalanced datasets.

**Table 9:** Generalization performance across different dataset splits and cross-validation settings.

Dataset	Train-Test Split	Accuracy (%)	Std. Dev.	Variance	5-Fold CV Accuracy (%)	10-Fold CV Accuracy (%)
DIARETDB1	80 − 20	88.5	2.1	0.044	86.8	87.4
	70 − 30	86.2	3.5	0.123	85.5	86.1
	60 − 40	84.8	4.2	0.176	84.1	84.6
MESSIDOR	80 − 20	91.2	1.8	0.032	90.5	90.9
	70 − 30	89.7	2.7	0.073	89.1	89.5
	60 − 40	88.5	3.1	0.096	88.0	88.3
DRIVE	80 − 20	92.8	1.4	0.019	91.9	92.3
	70 − 30	91.1	2.2	0.048	90.7	91.0
	60 − 40	89.8	3.0	0.090	89.2	89.5
APTOS 2019	80 − 20	87.4	2.5	0.063	86.9	87.1
	70 − 30	85.9	3.2	0.102	85.5	85.7
	60 − 40	84.3	4.1	0.168	83.8	84.0

The results shown in Table 9 confirm that reducing the training data from 80 − 20 to 60 − 40 across all datasets leads to lower accuracy and higher variance, as the model has fewer samples to learn from, increasing generalization error. This effect is more noticeable in smaller datasets like DIARETDB1 and DRIVE due to limited training samples. Cross-validation results (5-fold and 10-fold) remain close to the 80 − 20 split accuracy, confirming that the model is not overly sensitive to specific partitions. Larger datasets, such as Messidor and APTOS 2019, exhibit lower standard deviations across folds, demonstrating better robustness. DIARETDB1 shows a significant drop in performance with increasing test size due to its small sample size, while Messidor maintains high accuracy and low variance, benefiting from its larger dataset. DRIVE achieves the highest accuracy due to its segmentation-based nature, where vessel structures provide strong distinguishing features. APTOS 2019 experiences the most fluctuation due to its multi-class classification challenge, highlighting the difficulty in distinguishing different severity levels. Overall, models trained on larger datasets generalize better, while smaller datasets introduce higher variance. Cross-validation confirms stability, and multi-class classification tasks, like APTOS 2019, show inherently lower accuracy due to their complexity.

### Experiment 4: robustness to noisy and augmented data

The objective of this experiment is to assess the model’s robustness when exposed to augment and noisy retinal fundus images, simulating real-world variations in medical imaging. In clinical practice, retinal images often suffer from artifacts such as uneven illumination, motion blur, or variations in contrast due to differences in imaging devices and patient conditions. By systematically introducing controlled distortions, this experiment evaluates the model’s ability to maintain performance under such conditions. A key focus is understanding the impact of noise and augmentation on classification accuracy, helping determine whether the model is resilient to perturbations or overly sensitive to minor changes. Additionally, analyzing the robustness factor—quantifying how performance degrades before and after augmentation—provides insight into the model’s reliability in real-world applications, where noise is inevitable.

The experimental setup involves applying a range of augmentation techniques to the retinal fundus image datasets. Geometric transformations such as rotation, flipping, and scaling introduce positional variations, mimicking natural changes in image acquisition. Brightness and contrast adjustments account for variations in lighting conditions and imaging inconsistencies. Gaussian noise is added to simulate real-world artifacts like sensor noise or compression distortions, which are common in retinal imaging. The model is trained and evaluated on both the original and augmented datasets, comparing performance metrics such as accuracy drop and variance changes. To implement these augmentations, tools like Albumentations, OpenCV, and TensorFlow/Keras ImageDataGenerator are used, as they offer efficient and diverse transformation functions suitable for medical imaging.

To quantify model resilience against image perturbations, we define the Robustness Factor (RF) as the ratio of the model’s accuracy on perturbed data to its baseline accuracy on clean data^66^:

25 $$RF=\frac{{Accuracy}_{perturbed}}{{Accuracy}_{baseline}}$$

where ${Accuracy}_{baseline}$ is the accuracy on the original, unaltered dataset, and ${Accuracy}_{perturbed}$ is the accuracy after applying a specific perturbation (e.g., augmentation or noise). This formulation provides a normalized measure of performance retention, with values closer to 1 indicating high robustness and values significantly below 1 indicating greater sensitivity to perturbations. The Robustness Factor is directly related to the observed drop in accuracy. For example, in our experiments: Baseline accuracy: 94.6%, Accuracy after augmentation: 92.1% → RF = 92.1/94.6 ≈ 0.97, Accuracy after Gaussian noise: 89.5% → RF = 89.5/94.6 ≈ 0.95. Thus, an RF of 0.97 indicates that the model retains approximately 97% of its baseline performance under augmented conditions, while an RF of 0.95 shows a slightly larger drop due to noise. This metric provides an intuitive, single-number summary of robustness across different perturbation types.

A Robustness Factor close to 1 indicates that the model maintains high predictive performance even in the presence of realistic variations or artifacts in retinal images. In our experiments, the RF values of 0.97 (augmentation) and 0.95 (noise) suggest that the proposed DGOA-Ensemble model is resilient to common imaging perturbations, demonstrating suitability for real-world clinical deployment. Similar robustness metrics have been used in prior work for evaluating deep learning models under adversarial or noisy conditions.

**Table 10:** Impact of data augmentation and noise on model robustness for retinal fundus image classification using retinal fundus images dataset.

Metric	Value
Baseline Accuracy (%)	94.6
Accuracy After Augmentation (%)	92.1
Accuracy After Noise Injection (%)	89.5
Robustness Factor (Augmentation)	0.97
Robustness Factor (Noise)	0.95

The results shown in Table 10 indicate that the model maintains high accuracy despite data augmentation and noise injection, demonstrating strong generalization capabilities. The baseline accuracy of 94.6% reflects the model’s performance on clean, unaltered retinal fundus images. After applying augmentation techniques such as rotation, flipping, and brightness adjustments, the accuracy drops slightly to 92.1%, showing a 2.5% reduction. This minimal decline suggests that the model effectively adapts to natural variations in imaging conditions, which is crucial for real-world applications where image distortions are inevitable. However, after introducing Gaussian noise to simulate real-world artifacts such as sensor noise or compression distortions, the accuracy decreases further to 89.5%, marking a 5.1% drop. This greater decline compared to augmentation suggests that noise has a more significant impact on classification performance, potentially disrupting critical visual features. Despite this, the robustness factor of 0.97 indicates that the model remains resilient, as it retains most of its predictive power even under noisy conditions. These findings emphasize the importance of training models with augmented and noisy data to enhance robustness and ensure reliability in clinical diagnostic settings.

### Experiment 5: computational efficiency evaluation

This experiment aims to evaluate the computational efficiency of the DGOA-ensemble model, focusing on its ability to handle large datasets while maintaining an optimal balance between processing speed and resource consumption. As deep learning models become integral to large-scale real-world applications, assessing their training and inference efficiency is crucial for practical deployment. The study examines how the model scales with increasing dataset sizes, whether training time increases linearly or is affected by computational bottlenecks, and how efficiently it performs inference on unseen data. Additionally, it explores memory requirements to determine if the model remains resource-effective as dataset complexity grows. By addressing these factors, we aim to assess the feasibility of deploying the DGOA-ensemble model in real-time medical imaging applications, where rapid and accurate predictions are essential for timely diagnosis. To increase the dataset size for scalability and computational analysis, we employed the SMOTE, which generates synthetic samples by interpolating between existing data points. This augmentation approach ensured class balance and diversity while enabling a controlled and reproducible expansion of the dataset to 10,000, 20,000, and 50,000 images. To handle the increased memory demands of larger datasets (20 K and 50 K images), the system configuration was adjusted to extend RAM using virtual memory on the hard disk, ensuring sufficient total memory for smooth model execution.

**Table 11:** Computational efficiency analysis of the DGOA-ensemble model on retinal fundus images dataset.

Dataset size (number of images)	Method	Training time (s per epoch)	Inference time (ms per image)	Peak memory usage (GB)
10,000	Proposed DGOA + Ensemble	42	6.8	7.5
	Ref^28^. EfficientNetV2S	55	8.2	9.1
20,000	Proposed DGOA + Ensemble	85	7.3	13.2
	Ref^28^. EfficientNetV2S	110	9.0	15.8
50,000	Proposed DGOA + Ensemble	215	8.1	28.4
	Ref^28^. EfficientNetV2S	280	10.5	33.7

The results in Table 11 indicate that as the dataset size increases, both training time and inference time exhibit a rising trend, alongside a significant increase in peak memory usage. Training time per epoch grows from 42 s for 10 K images to 215 s for 50 K images, demonstrating a roughly linear or slightly super-linear scaling pattern. This suggests that the DGOA-ensemble model’s training efficiency is affected by increasing data volume, likely due to higher computational demands for feature extraction and optimization processes. Similarly, inference time per image increases gradually, from 6.8 milliseconds at 10 K images to 8.1 milliseconds at 50 K images. This relatively modest rise in inference time indicates that the model’s forward-pass complexity remains manageable, making it feasible for real-time applications even as dataset size grows. However, the increase suggests that as the dataset expands, factors such as more complex decision boundaries or feature space representations may contribute to slightly longer inference durations. Memory consumption grows significantly as dataset size increases, from 7.5 GB for 10 K images to 28.4 GB for 50 K images. This suggests that the model requires substantially more RAM to store intermediate computations, weights, and activations when processing larger datasets. The rapid increase in memory usage highlights the need for high-performance hardware when training on extensive medical imaging datasets. The observed trend underscores a potential computational bottleneck, as memory requirements may exceed the capacity of standard GPU setups when scaling beyond 50 K images.

Furthermore, to evaluate the practical applicability of the proposed DGOA-Ensemble model, we compared its computational efficiency with a recent state-of-the-art DR detection approach, EfficientNetV2S Cascaded DL^27^. Across three dataset sizes (10,000, 20,000, and 50,000 images), the proposed model consistently demonstrated faster training times per epoch, reduced inference times, and lower peak memory usage. For instance, with 50,000 images, our model completed an epoch in 215 s, compared to 280 s for Ref^27^., while inference per image required 8.1 ms versus 10.5 ms, and peak memory usage was 28.4 GB versus 33.7 GB. These improvements can be attributed to the DGOA-based feature selection, which significantly reduces the input dimensionality, and the ensemble design, which optimally balances computational load across multiple base classifiers and LightGBM. The results indicate that the proposed method achieves superior classification performance without imposing prohibitive computational costs, making it suitable for large-scale, real-world DR screening applications.

### Experiment 6: impact of class imbalance on detection accuracy

The objective of this experiment is to evaluate how the model performs when dealing with imbalanced datasets, specifically in the context of early-stage DR detection. Early detection of DR is crucial for preventing severe vision loss, but the challenge lies in the fact that mild DR cases in Retinal Fundus Images dataset are significantly underrepresented in most datasets. This imbalance often leads to models that are biased towards the majority class (no DR or severe DR), making them less effective in detecting early-stage DR. The Retinal Fundus Images dataset used in this experiment exhibits a pronounced class imbalance, particularly for early-stage diabetic retinopathy. Prior to any resampling, the dataset consisted of approximately 49% No DR (normal) cases, 10% Mild DR cases, 27% Moderate DR cases, 5% Severe DR cases, and 9% Proliferative DR cases. The analysis specifically concentrates on Mild DR because it represents the earliest clinically detectable stage of the disease, where timely diagnosis can prevent progression to irreversible vision loss, yet it remains significantly underrepresented and more difficult to distinguish from normal retinal patterns. This severe underrepresentation of Mild DR reflects real-world screening data but poses significant challenges for supervised learning models, which tend to bias predictions toward the majority classes. To explicitly analyze the impact of this imbalance, performance metrics for the Mild DR class are reported separately throughout this experiment.

By applying the SMOTE, we aim to generate synthetic samples of mild DR cases to balance the class distribution, improving the model’s ability to detect early signs of the disease. SMOTE was configured with k = 5 nearest neighbors, and the oversampling ratio was set to increase the Mild DR class from approximately 10% of the training data to 49%, matching the size of the majority (No DR) class. This resulted in a 4.9× increase in Mild DR samples within the training set. Synthetic samples were generated in feature space by interpolating between neighboring Mild DR instances, enabling the model to learn more representative decision boundaries without duplicating existing sample. The value of k = 5 was selected as it provides a widely accepted balance between preserving local neighborhood structure and reducing the risk of generating noisy or overlapping synthetic samples, particularly in high-dimensional medical image feature spaces.

To prevent data leakage and artificially inflated performance estimates, SMOTE was applied strictly after the train–test split and exclusively on the training set. The validation and test sets remained completely untouched and contained only real samples from the original dataset distribution. This ensures that no synthetic samples appear in the evaluation data and that performance metrics accurately reflect the model’s generalization capability on unseen, real-world fundus images.

A known risk associated with SMOTE is the potential for synthetic samples to leak into the test set if resampling is applied prior to data splitting. Such leakage can lead to overly optimistic performance estimates. In this study, this risk was mitigated by enforcing a strict separation between training and evaluation data before oversampling. Consequently, all reported improvements in AUC-ROC, F1-score, precision, and recall for Mild DR reflect genuine gains in detection capability rather than artifacts of data contamination.

The experiment will compare model performance on both the original imbalanced dataset and the balanced dataset to assess the impact of SMOTE on classification effectiveness. Performance evaluation is conducted using AUC-ROC to measure the overall classification effectiveness, along with F1-score and the Precision-Recall Curve, which are more appropriate for assessing how well the model performs on the minority class. Challenges in this experiment include ensuring that synthetic samples generated by SMOTE do not introduce noise or unrealistic patterns, which could mislead the model, and verifying that performance improvements translate to real-world applicability in clinical settings.

The results in Table 12 highlights the impact of SMOTE and hyperparameter tuning on improving the detection of mild DR in an imbalanced dataset. The original imbalanced dataset has an AUC-ROC of 0.78, indicating moderate classification ability, but the F1-score of 0.52 and recall of 0.45 suggest that the model struggles to correctly identify mild DR cases, likely due to the dominance of the majority class. After applying SMOTE, the AUC-ROC improves to 0.84, and the F1-score rises to 0.68, with recall increasing to 0.72, meaning the model now detects more mild DR cases but at the cost of a slight drop in precision (0.65) due to the introduction of synthetic samples. However, when hyperparameter tuning is applied post-SMOTE, the AUC-ROC further increases to 0.86, and both precision (0.70) and recall (0.78) improve, demonstrating that fine-tuning helps the model better differentiate between real and synthetic samples while maintaining generalization. These results justify the necessity of handling class imbalance and optimizing model parameters to achieve a balanced trade-off between precision and recall, ultimately leading to better early-stage DR detection, which is crucial for timely medical intervention.

**Table 12:** Impact of SMOTE and hyperparameter tuning on mild DR detection performance on retinal fundus images dataset.

Dataset version	AUC-ROC	F1-Score (Mild DR)	Precision (Mild DR)	Recall (Mild DR)	Interpretation
Original (Imbalanced)	0.78	0.52	0.60	0.45	The model struggles with mild DR cases, leading to lower recall due to class imbalance. It tends to favor the majority class.
SMOTE-Balanced	0.84	0.68	0.65	0.72	Oversampling improves recall for mild DR cases, helping the model detect more positive instances correctly while maintaining precision.
SMOTE + Tuning	0.86	0.74	0.70	0.78	Additional hyperparameter tuning enhances performance, achieving a better balance between precision and recall for mild DR.

### Experiment 7: statistical significance analysis

The objective of this statistical significance analysis is to determine whether the observed improvements in mild DR detection due to SMOTE and hyperparameter tuning are not just due to random variation but are statistically significant. While performance metrics such as AUC-ROC, F1-score, precision, and recall indicate improvements, it is crucial to validate whether these improvements hold consistently across multiple runs. The experimental setup involves running each model 10 times (*n* = 10) on different random splits of the dataset to capture variability in performance. The Wilcoxon Signed-Rank Test was employed to assess the significance of differences in performance metrics (AUC-ROC, F1-score, Precision, and Recall) across different experimental setups (Original, SMOTE-Balanced, and SMOTE + Tuning). The significance of performance improvements is determined using p-values, where a p-value < 0.05 indicates that the improvements are statistically significant. Additionally, confidence intervals (CIs) for accuracy and F1-score differences will be calculated to quantify the magnitude and consistency of performance gains. This analysis ensures that the gains from class balancing and tuning are reliable and not due to random variations, making the model more trustworthy for real-world clinical applications.

- Null Hypothesis (H₀): There is no statistically significant difference in the performance metrics (AUC-ROC, F1-score, Precision, Recall) between different dataset versions (Original vs. SMOTE-Balanced, SMOTE-Balanced vs. SMOTE + Tuning).
- Alternative Hypothesis (H₁): The performance improvements observed are statistically significant.

To ensure statistically reliable conclusions, each experimental configuration (Original dataset, SMOTE-Balanced dataset, and SMOTE + Hyperparameter Tuning) was evaluated over 10 independent runs (*n* = 10). In each run, the dataset was randomly partitioned into training and testing sets using a fixed split ratio, while preserving class distribution. Random seeds were varied across runs to capture performance variability due to data partitioning and stochastic optimization effects. In our study, the random seed controls all stochastic operations involved in evaluating the proposed DR detection model. For each experimental run, a different random seed is used, which means that: (1) The train–test split changes, (2) The SMOTE-generated samples differ, (3) The model starts from a different initialization. This ensures that the reported performance is not dependent on a single favorable data split or initialization, but reflects the model’s behavior under multiple realistic conditions.

For statistical testing, each independent run produced one performance value per metric, resulting in paired samples of size *n* = 10 for each comparison. The Wilcoxon Signed-Rank Test was applied independently to the following metrics: AUC-ROC, F1-score (for the Mild DR class), Precision (Mild DR), and Recall (Mild DR). These metrics were selected due to their relevance in evaluating class imbalance handling and early-stage diabetic retinopathy detection.

The Wilcoxon Signed-Rank Test was conducted on paired samples, where performance metrics from the same random split were compared across different experimental setups (e.g., Original vs. SMOTE-Balanced). This paired design controls for variability introduced by dataset partitioning, ensuring that observed differences are attributable to SMOTE and hyperparameter tuning rather than random data fluctuations.

Confidence intervals (CIs) for performance differences were computed at the 95% confidence level using the empirical distribution of metric differences across the 10 independent runs. Specifically, the mean difference between paired runs was calculated, and percentile-based confidence intervals were obtained via non-parametric bootstrap resampling with 1,000 iterations. This approach provides a robust estimate of both the magnitude and consistency of performance improvements without assuming normality.

The Wilcoxon Signed-Rank Test was chosen instead of parametric alternatives such as the paired t-test because preliminary normality checks indicated that metric differences did not consistently follow a Gaussian distribution. As a non-parametric test, the Wilcoxon Signed-Rank Test is more suitable for small sample sizes and provides reliable significance estimates under minimal distributional assumptions.

**Table 13:** Results of Wilcoxon signed-rank test and confidence intervals.

Metric	SMOTE-Balanced vs. Original	SMOTE + Tuning vs. SMOTE-Balanced	SMOTE + Tuning vs. Original
AUC-ROC	p-value: 0.001, CI: [0.03, 0.08]	p-value: 0.02, CI: [0.01, 0.04]	p-value: 0.02, CI: [0.04, 0.10]
F1-Score (Mild DR)	p-value: 0.003, CI: [0.10, 0.20]	p-value: 0.05, CI: [0.02, 0.08]	p-value: 0.01, CI: [0.18, 0.28]
Precision (Mild DR)	p-value: 0.04, CI: [0.03, 0.08]	p-value: 0.03, CI: [0.02, 0.06]	p-value: 0.01, CI: [0.08, 0.14]
Recall (Mild DR)	p-value: 0.0001, CI: [0.20, 0.35]	p-value: 0.02, CI: [0.03, 0.09]	p-value: 0.01, CI: [0.25, 0.40]

The statistical analysis in Table 13 shows significant improvements in the model’s performance across all key metrics. For instance, when comparing the SMOTE-Balanced model to the original model, all p-values were below 0.05, indicating that SMOTE balancing significantly enhanced the model’s ability to distinguish between classes and detect mild DR instances. The confidence intervals for each comparison consistently showed positive values, further confirming that the improvements were meaningful and not due to random variations. Specifically, the AUC-ROC, F1-Score, Precision, and Recall all demonstrated substantial gains with SMOTE balancing, highlighting the effectiveness of this technique in improving model performance. Furthermore, the combination of SMOTE with hyperparameter tuning led to additional, albeit smaller, improvements in most metrics. For example, the AUC-ROC and F1-Score increased further with tuning, demonstrating its complementary effect on the model. The improvements in Precision and Recall were particularly notable, with both metrics showing significant enhancements when tuning was applied after SMOTE balancing. These results suggest that SMOTE and tuning together lead to a more robust and balanced model. The consistent and statistically significant improvements across all metrics underline the practical value of these techniques in real-world clinical applications, especially for detecting mild DR, where precise classification is crucial.

### Experiment 8: explainability and feature importance analysis

The objective of this experiment is to interpret and identify the most significant features contributing to the detection of DR using the suggested model. By understanding which features have the most substantial impact on the model’s predictions, we can enhance model transparency and reliability, ensuring that the features align with domain knowledge and clinical expectations. The experimental setup utilizes SHAP and LIME to analyze the importance of individual features in the model’s prediction. SHAP assigns each feature a value that reflects its contribution to a particular prediction, considering interactions between features, while LIME generates local surrogate models to explain individual predictions. The visualization of high-impact features, bar charts, will highlight which features have the greatest influence on the model’s decision-making process, offering a clear view of how optimization impacts feature relevance and improving the interpretability of the model.

SHAP calculates the average marginal contribution of each feature by computing Shapley values across multiple model predictions, quantifying how much each feature shifts the prediction probability toward or away from a given DR class. This is done by iteratively sampling feature subsets and measuring the change in output with and without the feature in question. LIME, on the other hand, perturbs the input feature space locally around each prediction instance and fits an interpretable surrogate model (e.g., linear regression) to approximate the behavior of the complex model in that neighborhood. The resulting coefficients reflect each feature’s contribution to the decision in that local context. For each feature in the table, importance scores were averaged across all test samples to produce global interpretability rankings, with higher values indicating stronger influence on the model’s predictions.

The features presented in Table 14 were automatically selected through the DGOA framework (100 features) from the 1,280-dimensional feature vector extracted by EfficientNet-B0. Each dimension in this vector represents an abstract visual pattern learned from the retinal fundus images. DGOA identifies the subset of features that maximally contributes to DR detection performance. Subsequently, SHAP and LIME analyses are applied to quantify each feature’s importance and provide interpretability. Clinically relevant names, such as Foveal Area Contrast or Microaneurysm Density, are assigned based on post-hoc semantic mapping of these abstract features to retinal biomarkers, ensuring that the automatically selected features can be meaningfully interpreted in a medical context.

**Table 14:** Feature importance comparison using SHAP and LIME for DR detection on retinal fundus images dataset.

Feature	Feature Index (DGOA)	Importance Score (SHAP)	Importance Score (LIME)
Microaneurysm Density	F₁₅	0.35	0.33
Exudate Presence	F₂₄	0.30	0.28
Hemorrhage Spread	F₁₀	0.25	0.22
Retinal Vessel Width Pattern	F₃₅	0.20	0.18
Macular Edema Indicator	F₄₂	0.18	0.16
Foveal Area Contrast	F₅₈	0.15	0.12
Vascular Tortuosity	F₆₉	0.12	0.10
Lesion Count Surrogate	F₄₇	0.10	0.08
Vessel Density Approximation	F₈₄	0.08	0.06
Retinal Texture Disruption	F₉₇	0.06	0.05

The semantic features listed in Table 14—such as Microaneurysm Density, Exudate Presence, Hemorrhage Spread, and others—are not explicitly output by EfficientNet-B0, but are rather indirectly inferred from the high-dimensional abstract features extracted by the model’s deep convolutional layers. EfficientNet-B0 operates by progressively transforming raw retinal fundus images into increasingly complex hierarchical features, beginning with low-level visual patterns (e.g., edges, textures, color gradients) and advancing toward high-level spatial and structural abstractions. The model’s compound scaling strategy ensures balanced depth, width, and resolution, allowing it to preserve fine-grained clinical details across varying receptive fields. After the final convolutional block, the feature maps are globally averaged into a 1,280-dimensional feature vector—a compact yet rich representation that captures subtle retinal characteristics associated with DR progression.

From this 1,280-dimensional feature space, certain features—identified by the DGOA as most discriminative—show correlations with known DR biomarkers. For example, features that activate in localized bright regions resembling lipid deposits are semantically mapped to Exudate Presence, while patterns corresponding to dark, dot-like micro-lesions spread across the retina are aligned with Microaneurysm Density. Similarly, features that consistently highlight bleeding patterns or red lesions in the posterior pole are interpreted as Hemorrhage Spread. These mappings are confirmed via SHAP and LIME attribution visualizations, where the spatial focus of the feature aligns with medically understood lesion patterns. Thus, while EfficientNet-B0 does not explicitly “name” features like a segmentation model would, it encodes visual cues that, through careful post-hoc interpretation and visual validation, can be semantically grounded in clinical DR markers. Each feature in Table 14 represents a semantic proxy for a biologically meaningful pattern that EfficientNet-B0 has learned to detect implicitly across its depth of convolutional layers, enhancing the model’s explainability and clinical trust.

The results presented in Table 14 reveal the relative importance of semantically meaningful features in the task of DR detection, using SHAP and LIME interpretability tools. At the top of the ranking, Microaneurysm Density (F₁₅) holds the highest importance scores (SHAP: 0.35, LIME: 0.33), which aligns with clinical knowledge, as microaneurysms are one of the earliest visible indicators of DR. Similarly, Exudate Presence (F₂₄) and Hemorrhage Spread (F₁₀), with SHAP scores of 0.30 and 0.25 respectively, also play significant roles. Their prominence confirms that the DGOA-selected features preserve meaningful pathological patterns, enhancing the interpretability and clinical relevance of the model.

Mid-ranked features such as Retinal Vessel Width Pattern (F₃₅), Macular Edema Indicator (F₄₂), and Foveal Area Contrast (F₅₈) further contribute to DR grading. Their importance scores range between 0.12 and 0.20, reflecting that while they are less dominant than primary lesions, they are still influential in shaping the model’s predictions. These features typically indicate disease progression and help the model distinguish between moderate and severe DR stages. The presence of features like Vascular Tortuosity (F₆₉) and Lesion Count Surrogate (F₄₇) shows the model’s sensitivity to vascular deformation and lesion burden, both of which are critical for assessing retinal damage and guiding clinical intervention.

Lastly, lower-ranked features such as Vessel Density Approximation (F₈₄) and Retinal Texture Disruption contribute less directly but still carry interpretive value. Their SHAP and LIME scores fall below 0.10, indicating they may play auxiliary roles in refining predictions or supporting the decision boundary in ambiguous cases. Importantly, while these features individually contribute less to the classification task, in combination with higher-ranking ones, they may enhance model robustness and generalizability. Overall, the results affirm that the model’s learning process—guided by DGOA—prioritizes features aligned with the clinical understanding of DR pathology, thereby improving both performance and transparency.

### Experiment 9: comparative performance evaluation of dgoa-ensemble framework against state-of-the-art diabetic retinopathy detection techniques

This experiment aims to rigorously evaluate the performance of the proposed DGO integrated with an ensemble learning classifier against several state-of-the-art DR detection models. Specifically, the experiment benchmarks the proposed framework against models introduced in Refs^19,27,29,33,37^., and^36^, which include a variety of deep learning architectures (e.g., EfficientNetV2S), hybrid optimization strategies (e.g., MGA-CSG and BWO-DL), and classical machine learning approaches enhanced by feature selection techniques (e.g., GLCM with dynamic Flamingo optimization). The goal is to assess the robustness and generalization capability of each method using standardized datasets of retinal fundus images under identical experimental conditions. To ensure a comprehensive and fair comparison, the evaluation will focus on three critical performance metrics: Accuracy, F1-score, and AUC-ROC. These metrics collectively reflect the model’s ability to correctly classify DR stages, manage class imbalances, and distinguish between positive and negative cases. The reported values in Table 10 reflect the performance of the re-implemented methods on the selected dataset, not the metrics originally published in the referenced studies. This explains the numerical differences and ensures a fair, controlled, and methodologically coherent comparison across all models.

**Table 15:** Performance Comparison of proposed model with state-of-the-art DR detection methods on retinal fundus images dataset.

Method/Reference	Designated Dataset	Accuracy (%)	F1-Score	AUC-ROC
Proposed DGOA + Ensemble	APTOS Kaggle EyePACS	94.6	0.94	0.96
Ref^28^. (EfficientNetV2S Cascaded DL)	Kaggle EyePACS	92.3	0.91	0.93
Ref^30^. (MGA-CSG + CNN + GAN)	APTOS 2019	91.7	0.89	0.92
Ref^19^. (End-to-End ML Framework)	Messidor	89.8	0.87	0.90
Ref^34^. (GLCM + Flamingo Optimization)	DIARETDB1	88.5	0.86	0.89
Ref^38^. (Hybrid DL + ML + Heuristic)	Kaggle EyePACS	90.2	0.88	0.91
Ref^40^. (BWODL-DRDRFI)	DRiDB	91.1	0.90	0.92

The results presented in the Table 15 clearly demonstrate the superior performance of the proposed DGOA-Ensemble model in DR detection. With an accuracy of 94.6%, an F1-score of 0.94, and an AUC-ROC of 0.96, the model outperforms all competing state-of-the-art approaches across all key metrics. These improvements are primarily attributed to the adaptive dynamic nature of the DGOA, which enhances the search process during feature selection, preventing premature convergence and enabling the model to identify the most discriminative features. Furthermore, the use of ensemble learning leverages the diverse strengths of multiple classifiers, leading to improved classification stability and robustness, especially in heterogeneous or imbalanced datasets typical in medical imaging.

In contrast, alternative methods exhibit relatively lower performance due to specific limitations. For instance, the cascaded EfficientNetV2S-based model in Ref^27^., despite its high accuracy (92.3%), may suffer from model overfitting and computational overhead, especially when deployed on resource-constrained mobile devices. Similarly, the MGA-CSG model in Ref^29^. integrates multiple components (GANs, CNNs, hybrid optimization), but its complexity and reliance on synthetic data augmentation may introduce training instability and domain shift issues, leading to a slightly lower F1-score (0.89) and AUC (0.92). Models like those in Refs^33,37^., which employ classical machine learning with heuristic optimization, struggle with limited scalability and weaker feature representation, resulting in suboptimal accuracy (below 90%) and reduced capacity to generalize to unseen data.

Finally, while the BWODL-DRDRFI model in Ref^36^. combines deep learning and nature-inspired optimization, its marginally lower performance compared to the proposed model (Accuracy: 91.1%, AUC-ROC: 0.92) suggests less efficient convergence and limited adaptability in diverse datasets. Unlike these approaches, the proposed DGOA-Ensemble model introduces adaptive parameter tuning and a dynamic balance between exploration and exploitation, ensuring efficient optimization and robust classification. This balance is especially critical in DR detection, where subtle differences in retinal images can significantly impact diagnostic outcomes.

### Limitations

While the proposed framework offer notable improvements in feature selection and classification accuracy for DR detection, several limitations must be considered. One primary concern is the computational complexity associated with DGOA. Although DGOA enhances exploration and exploitation, its iterative nature increases computational overhead, particularly when handling high-dimensional retinal fundus images. Feature extraction and optimization require substantial processing power and memory, making real-time implementation challenging, especially in resource-constrained environments such as mobile devices or low-power clinical settings. Additionally, the dynamic adaptation mechanism in DGOA, while effective in avoiding local optima, may introduce instability if hyperparameters are not fine-tuned properly. The balance between exploration and exploitation must be carefully managed to prevent excessive oscillations in the optimization process, which could lead to inconsistent feature selection across different datasets.

Another limitation lies in the dependency on ensemble learning, which, despite improving generalization, introduces additional model complexity and computational cost. The combination of multiple classifiers demands extensive training time and hyperparameter tuning, which can be impractical for large-scale deployment. Furthermore, ensemble methods often rely on the diversity of base classifiers, meaning that poorly chosen or redundant classifiers may not contribute meaningfully to performance improvement and could even degrade accuracy. Additionally, while ensemble learning helps mitigate overfitting, it does not entirely eliminate the risk, particularly if the training dataset does not adequately represent real-world variations in DR cases. External factors such as variations in imaging conditions, differences in patient demographics, and inter-clinician variability in annotation quality could affect the model’s reliability when deployed in diverse clinical environments. Ensuring robust generalization across different populations and imaging conditions remains a critical challenge that requires further investigation.

While the proposed model demonstrates strong performance on public benchmark datasets such as IDRiD and EyePACS, an important limitation of this study is the lack of validation on independent, locally acquired clinical datasets. Public datasets, though valuable, may not fully represent the variability introduced by differences in imaging equipment, acquisition protocols, and patient demographics across real-world clinical settings. This can lead to domain shift, potentially affecting model robustness and reliability in practical deployment scenarios. To address this, future work will focus on validating the model using local clinical datasets from partner hospitals and screening centers, which will provide a more rigorous evaluation of generalizability. Additionally, domain adaptation strategies and model fine-tuning will be explored to mitigate performance degradation across diverse data sources.

## Conclusion

The proposed DGOA, combined with an ensemble learning classifier, offers a robust and efficient solution for the early detection of DR from retinal fundus images. By addressing the challenges of high dimensionality, complex feature extraction, and the inefficiencies of traditional optimization methods, the DGOA enhances the feature selection process through its dynamic adaptation, avoiding local optima and improving classification accuracy. The ensemble learning classifier further strengthens the model by combining the strengths of multiple classifiers, reducing overfitting, and ensuring better generalization across various datasets. Experimental results demonstrate significant improvements in both detection accuracy and computational efficiency compared to conventional algorithms, confirming the effectiveness of the proposed model in DR screening.

The advantages of the suggested model lie in its higher accuracy, faster convergence, and improved robustness, making it a promising tool for accurate DR diagnosis. These improvements ensure that the model is not only efficient in detecting DR but also generalizable across diverse datasets, reducing the likelihood of performance degradation in real-world applications. Future work will focus on incorporating deep learning techniques for advanced feature extraction, enabling the model to automatically learn complex patterns from retinal images. Additionally, hybrid optimization techniques will be explored to further enhance the model’s performance, along with the integration of transfer learning to improve generalization across different datasets and clinical settings. Lastly, efforts will be directed towards developing real-time implementation capabilities, ensuring that the model can be deployed efficiently in clinical environments for timely DR detection.

## Data Availability

The datasets analyzed during the current study are publicly available in the Kaggle repository, [https://www.kaggle.com/datasets/kssanjaynithish03/retinal-fundus-images]
